# Synergetic Insulation and Induction Effects Selectively Optimize Multiresonance Thermally Activated Delayed Fluorescence

**DOI:** 10.34133/2022/9838120

**Published:** 2022-06-02

**Authors:** Jinkun Bian, Su Chen, Lili Qiu, Nan Zhang, Jing Zhang, Chunbo Duan, Chunmiao Han, Hui Xu

**Affiliations:** Key Laboratory of Functional Inorganic Material Chemistry, Ministry of Education, School of Chemistry and Materials, Heilongjiang University, China

## Abstract

Multiresonance (MR) emitters featuring narrowband emissions and theoretically 100% exciton harvesting are great potential for organic light-emitting diode (OLED) applications. However, how to functionalize MR molecules without scarifying emission color purity is still a key challenge. Herein, we report a feasible strategy for selective optimization of MR molecules, which is demonstrated by a blue MR emitter **tCBNDASPO** substituted with a diphenylphosphine oxide (DPPO) group. Compared to its DPPO-free parent molecule, **tCBNDASPO** preserves narrowband feature with full widths at half maximum (FWHM) values of 28 nm in film and 32 nm in OLEDs and achieves 40% increased photoluminescence (92%) and electroluminescence quantum efficiencies (28%). It is showed that insulation effect of P=O effectively confines the singlet excited state on MR core to keep emission color purity, and its induction effect enhances singlet radiation and triplet-to-singlet conversion. This synergism for selective optimization is based on rational linkage between MR core and functional groups.

## 1. Introduction

For high-resolution photonic applications, emission color purity is one of the most important properties of luminescent materials and devices, which requires narrow full width at half maximum (FWHM) of emission peak [1]. Organic molecules feature relatively broadband emission characteristic of multiple vibrational transitions [2]. It is known that flexible moieties and intramolecular charge transfer (ICT) between donor (D) and acceptor (A) groups can significantly increase vibrational levels and widen spectral profiles, rendering FWHM > 100 nm [3]. In contrast, polycyclic aromatic hydrocarbon, such as anthracene and pyrene, shows advantageous FWHM values within 50 nm, owing to their highly rigid structures and locally excited (LE) first singlet states (^1^LE) [4]. Nevertheless, fluorescent characteristics and simple electrical properties of hydrocarbons limit their performance for photonic applications, e.g., organic light-emitting diodes (OLEDs) [5–12]. In recent years, thermally activated delayed fluorescence (TADF) featured pure-organic materials rapidly emerged for OLED applications, owing to the merits of 100% theoretical internal quantum efficiency (IQE, *η*_IQE_), low cost, and high sustainability [13–16]. Singlet-triplet splitting energies (Δ*E*_ST_) of TADF molecules are nearly zero, so that nonradiative triplet excitons can be upconverted to radiative singlet excitons, through reverse intersystem crossing (RISC). Because Δ*E*_ST_ is directly proportional to overlap integral of frontier molecular orbitals (FMO), D-A structure is most widely adopted [17, 18]. As consequence, the charge transfer-featured first singlet excited states (^1^CT) of TADF materials render FWHM around 100 nm, leading to unsatisfied color purities of TADF OLEDs.

**Scheme 1:**
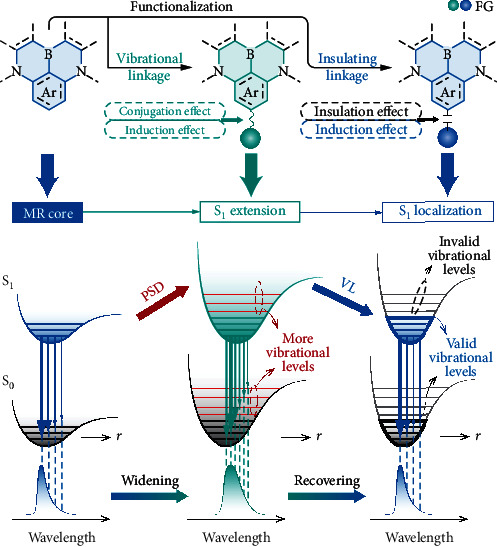
Influence of functionalization on emission properties of multiresonance (MR) emitters and design proposal of “insulating induction” strategy. The incorporation of the functional groups (FG) in the first singlet states (*S*_1_) renders potential surface deepening (PSD) and emission bathochromic shift and widening, due to involving in more vibrational levels. The insulating linkage between MR core and FG prevents the *S*_1_ extension. Therefore, the *S*_1_ state is confined on MR core through vibrational limitation (VL), inducing recovered narrow emission band.

In 2016, Hatakeyama et al. reported a new class of TADF-featured polycyclic aromatics, namely, multiresonance (MR) TADF emitters, e.g., **DABNA-1**, whose FWHM values were ~30 nm [19, 20]. In MR molecules, instead of the D-A groups, resonance effects of electron withdrawing and donating atoms, e.g., boron and nitrogen, are used to separate FMOs [21–26]. Therefore, high structural rigidity and effective ICT can be integrated to realize narrowband TADF emission [23]. Through conjugation extension and functional modification, blue [27–33], green [34–39], yellow [40, 41], and red [42] TADF emitters were developed, whose device efficiencies were comparable to those of the most efficient counterparts, e.g., the maximum external quantum efficiencies (EQE, *η*_EQE_) >20%. Modifying MR core with the electroactive D and A groups can markedly improve device performance but simultaneously induce bathochromic shifts and increased FWHM (>40 nm). It shows that conjugated bonding with the D/A groups induces the combined conjugation and induction effects on MR cores, which deepen potential energy surfaces of the first singlet excited states (*S*_1_), respectively, due to the additional vibrational levels contributed by the D/A groups and the involvement of intergroup charge transfer components in the *S*_1_ states (Scheme 1) [43].

**Figure 1:**
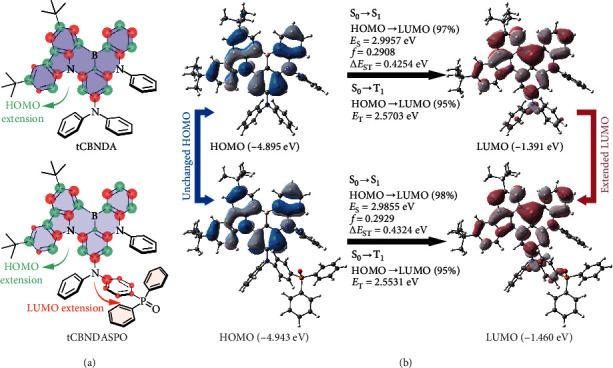
Chemical structures and electronic characteristics of **tCBNDA** and **tCBNDASPO**. (a) Chemical structures of **tCBNDA** and **tCBNDASPO**, whose MR cores and localized HOMO and LUMO distributions are highlighted with purple, green, and red colors, respectively. Green and red arrows indicate the HOMO and LUMO extensions, respectively, through conjugation extension and selective induction effect of diphenylphosphine oxide (DPPO), respectively. (b) Contours of the HOMOs and the LUMOs of **tCBNDA** and **tCBNDASPO** and transition parameters of their singlet and triplet excitations. Weights of HOMO→LUMO transitions are highlighted to reveal the charge transfer characters of their excited states. *f*, *E*_*S*_, and Δ*E*_ST_ refer to singlet oscillator strength, the *S*_1_ energy level, and singlet-triplet splitting energy, respectively.

Obviously, the negative influences of vibrational linkages on emission color purities of MR chromophores significantly limit functionalization and further performance improvement of MR TADF materials. So, a rational molecular design strategy should be based on avoiding the involvement of electron-withdrawing/donating groups in the *S*_1_ states but simultaneously utilizing their induction effects to optimize ICT in MR core. In this sense, insulating linkage provides a feasible way to combine color purity preservation and efficiency improvement, because it can (i) interrupt conjugation interactions with functional groups, therefore excluding them from the *S*_1_ state [44], and (ii) provide additional induction effect to accurately modulate ICT [45, 46] within MR chromophores (Scheme 1). Herein, as a proof of concept, we develop a blue MR TADF emitter named **tCBNDASPO**, which consists of a B-N MR framework, namely, the parent molecule **tCBNDA**, and a functional group of diphenylphosphine oxide (DPPO) (Figure 1(a) and Scheme S1). Owing to insulation effect of P=O linkage [47], the DPPO group is completely excluded from the *S*_1_ state. Simultaneously, induction effect of the P=O group extends the lowest unoccupied molecular orbital (LUMO) of **tCBNDASPO**, increasing FMO overlap and thereby singlet oscillator strength (*f*_*S*_). As expected, in addition to the identical single-molecular photoluminescence (PL) spectrum with the unchanged FWHM value of 28 nm, PL quantum yield (*ϕ*_PL_) of **tCBNDASPO** is improved by ~30% to 92%, owing to its 17-fold increased singlet radiation rate constant (*k*_*r*_^*S*^) and more than halved triplet nonradiation rate constant (*k*_*nr*_^*T*^). Consequently, **tCBNDASPO**-based blue OLEDs achieved the excellent color purity and the state-of-the-art *η*_EQE_ up to 28.0%, which was 40% higher than that of **tCBNDA**-based analog. Different to previous report [48], the comparison between MR emitters with/without phosphorylation clearly indicates synergism of insulation and induction effects on EL performance enhancement, which provides an effective way for selective functional extension and optimization of MR systems.

## 2. Results

### 2.1. Structures and Gaussian Simulation Results

Chemical structures of **tCBNDA** and **tCBNDASPO** were fully characterized with NMR and mass spectra and elemental analyses (see experimental section in supporting information). A direct borylation method [49] was used to inset boron atom at ortho-position between 3,6-di(*tert*-butyl)carbazole (tCz) and one diphenylamine (DPA) (Scheme S1). Density functional theory (DFT) calculation shows that due to its planar and conjugated structure, whole carbazole is fused in B-N framework (Figures S1-S5). As a result, the highest occupied molecular orbitals (HOMO) are extended to whole carbazole ring, in contrast to only one phenyl contributed by fused DPA group (Figure 1(b) and S1). It is noted that the HOMO locations of **tCBNDA** and **tCBNDASPO** are identical, indicating the exclusion of DPPO from direct D-A interactions. Furthermore, DPPO substitution induces the decreases of the HOMO and LUMO energy levels by only 0.05 and 0.07 eV, which are consistent with the same experimentally measured values of -5.7 and -3.0 eV for **tCBNDA** and **tCBNDASPO** (Figure S8 and Table S1). Nonetheless, the electron-withdrawing effect of P=O induces appropriate extension of the lowest unoccupied molecular orbital (LUMO) to nitrogen atom of diphenylamine of **tCBNDASPO**, thereby increasing probability of FMO overlap. Despite its negligible influences on occupied molecular orbitals, induction effect of P=O makes the LUMO+1 and the LUMO+2 shift from MR core to DPPO.

Natural transition orbital (NTO) investigation indicates that the *S*_0_⟶*S*_1_ and *S*_0_⟶*T*_1_ excitations of **tCBNDA** and **tCBNDASPO** are predominantly contributed by HOMO→LUMO transitions (weights > 90%) (Figure 1(b) and S2-S3). Thus, the *S*_1_ and *T*_1_ energy levels and Δ*E*_ST_ values of **tCBNDA** and **tCBNDASPO** are around 3.0, 2.6, and 0.43 eV, with negligible differences within 0.02 eV. Compared to **tCBNDA**, LUMO extension in **tCBNDASPO** leads to slightly increased overlap integrals of FMO wave functions (〈Ψ_*H*_|Ψ_*L*_〉) and electron cloud densities (〈Ψ_*H*_^2^|Ψ_*L*_^2^〉) but markedly shortened centroid-centroid distances of FMOs (*d*_*H*−*L*_) at the ground (*S*_0_), *S*_1_ and *T*_1_ states (Figure S4). As a result, the singlet oscillator strength (*f*_*S*_) of **tCBNDASPO** reaches to 0.2929, which is 0.0021 larger than that of **tCBNDA**. More importantly, the *S*_1_ state of **tCBNDASPO** is completely localized on its **tCBNDA** core. It indicates that although nonplanar DPPO could introduce additional vibrational levels, its steric hindrance actually restrains vibration of its linked phenyls, and insulation effect of P=O excludes it from excited-state transitions. In this case, valid vibrational levels involved in radiation are still thoroughly contributed by **tCBNDA** core (Scheme 1). Such vibrational limitation (VL) would support the consistence between **tCBNDA** and **tCBNDASPO** in emission profiles. Although the same FMO locations of the *S*_1_ and *T*_1_ excitations make spin-orbital coupling vanished, the second triplet (*T*_2_) states of **tCBNDA** and **tCBNDASPO** provide a feasible channel for effective RISC (Figures S5-S7).

### 2.2. Photophysical Properties

**Figure 2:**
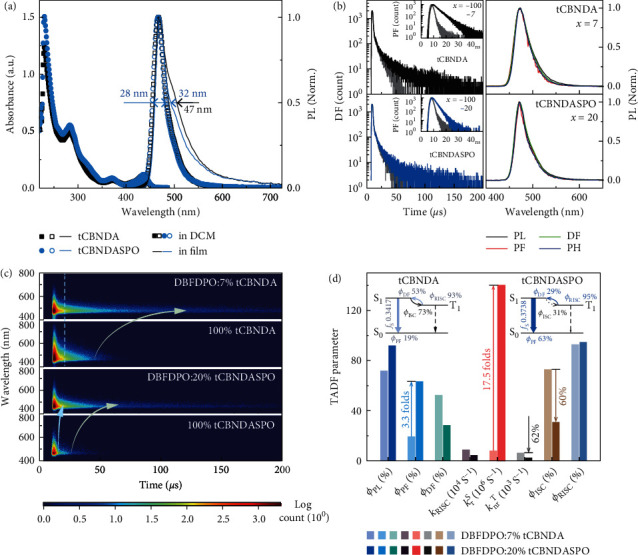
Photophyscial properties of **tCBNDA** and **tCBNDASPO**. (a) Electronic absorption and photoluminescence (PL) spectra of **tCBNDA** and **tCBNDASPO** in dilute dichloromethane solutions (10^−6^ mol L^−1^) and PL spectra of their *vacuum*-evaporated neat films (100 nm). (b) Time decay curves (left) and steady-state and transient emission spectra (right) of *vacuum*-evaporated DBFDPO:*x*% MR emitter films. For neat films, *x* = 100. PL, PF, DF, and pH refer to steady-state emission, prompt fluorescence, delayed fluorescence, and phosphorescence, respectively. PF, DF, and pH were recorded in ranges of 0-0.1 *μ*s, 1-100 *μ*s, and 100-200 *μ*s. (c) Transient emission contours of DBFDPO:*x*% MR emitter films. (d) Key TADF transition parameters of DBFDPO:*x*% MR emitter films. *η* and *k* refer to quantum efficiency and rate constant, respectively. Subscripts of ISC, RISC, *r*, and nr refer to intersystem crossing, reverse ISC, radiation, and nonradiation. Superscripts of *S* and *T* refer to singlet and triplet. The detailed transition processes of **tCBNDA** and **tCBNDASPO** are illustrated on left and right corners, respectively, in which *f* is experimental value of oscillator strength evaluated with electronic absorption.

In accord with time-dependent DFT (TDDFT) results, electronic spectra of **tCBNDA** and **tCBNDASPO** in dilute dichloromethane solutions (10^−6^ mol L^−1^) consist of exactly the same absorption bands, corresponding to *π*⟶*π*∗ (<300 nm), *n*⟶*π*∗ (300-400 nm), and charge-transfer (~450 nm) transitions (Figure 2(a) and Table S1). It indicates that DPPO is indeed not involved in the singlet excitation of **tCBNDASPO**. Moreover, experimentally estimated *f*_*S*_ of **tCBNDASPO** is as high as 0.3738, over 0.3417 of **tCBNDA**. PL spectra of these two molecules in dilute solutions completely overlap, corresponding to blue emissions with peak wavelengths at 467 nm. The solvatochromic properties of **tCBNDA** and **tCBNDASPO** are also the same (Figure S9). Therefore, DPPO does not significantly change ICT in the latter. As consequence, FWHM values of **tCBNDA** and **tCBNDASPO** in dilute dichloromethane are identical and as small as 28 nm, which are even smaller than 33 nm of **DABNA-1** [19] without functionalization.

In neat films, emission peaks of **tCBNDA** and **tCBNDASPO** slightly shift to 470 nm (Figure 2(a) and Table S1). However, FWHM of **tCBNDA** neat film markedly increases to 47 nm, due to its planar structure induced aggregation. In contrast, **tCBNDASPO** neat film still preserves a small FWHM of 32 nm, owing to its asymmetric structure and steric hindrance of DPPO. **tCBNDA** and **tCBNDASPO** are further dispersed in a host matrix 4,6-bis(diphenylphosphoryl)dibenzofuran (DBFDPO) [50] to form *vacuum*-evaporated films of DBFDPO:*x*% MR emitters. It is showed that concentration dependence of PL spectra for this two MR emitters is different (Figure S10). FWHM of DBFDPO:*x*% **tCBNDA** is linearly proportional to *x*%, reflecting serious self-aggregation tendency (Figure S11). Furthermore, *ϕ*_PL_ of **tCBNDA**-based films reaches the highest value of 72% at a low *x* = 7, then rapidly decreases to 29% at *x* = 20. In contrast, FWHM of DBFDPO:*x*% **tCBNDASPO** (32 nm) is independent on *x*. **tCBNDASPO** simultaneously endows its films with the highest *ϕ*_PL_ of 92% at *x* = 20. It indicates that steric hindrance of DPPO effectively suppresses self-aggregation and alleviates intermolecular interaction-induced quenching.

PL decays of **tCBNDA**- and **tCBNDASPO**-based films consist of ns-scale prompt fluorescence (PF) and *μ*s-scale delayed fluorescence (DF) components (Figures S12 and S13). It is showed that DBFDPO matrix can effective restrain concentration quenching. Therefore, PF (*τ*_PF_) and DF (*τ*_DF_) lifetimes are in reverse proportion to *x*% (Table S2). Nevertheless, emission lifetimes of **tCBNDA** are more dependent on *x*%, in accord with its stronger intermolecular interactions. Compared to neat film, *τ*_PF_ and *τ*_DF_ of DBFDPO:7% **tCBNDA** are markedly increased by 2.5 folds, respectively. In contrast, DBFDPO:20% **tCBNDASPO** reveals 1.4 folds and 0.3 fold increased *τ*_PF_ and *τ*_DF_, respectively. It is known that triplet-involved DF with longer lifetime should suffer from more serious quenching. However, compared to its PF, when *x* ≥ 20, DF of DBFDPO:*x*% **tCBNDASPO** is unexpectedly less sensitive to doping concentration, reflecting highly efficient RISC and reduced triplet quenching.

DF intensities of **tCBNDA**- and **tCBNDASPO**-based films are in reverse proportion to temperature (Figures S14 and S15), corresponding to transition from *T*_1_-originated phosphorescence (pH) to the *S*_1_ state with markedly larger allowedness through thermally activation. Temperature-dependent time resolved emission spectra (TRES) show the same tendencies (Figures S16 and S17). PF, DF, and pH spectra of **tCBNDA** and **tCBNDASPO** are nearly overlapped, corresponding to near-zero Δ*E*_ST_ values of 0.07 and 0.05 eV, respectively (Figure 2(b) and Table S1). TRES of DBFDPO:*x*% MR emitters indicate that DF components are in reverse proportion to *x*%, since the sensitivity of triplet states to concentration quenching (Figure S18). Furthermore, compared to neat film, DF component of DBFDPO:7% **tCBNDA** is more significantly enhanced than its PF component. Therefore, triplet involved processes, e.g., Dexter energy transfer (DEXT) and triplet quenching, are main factors influencing emission properties of **tCBNDA**-based films. In contrast, both PF and DF components of DBFDPO:20% **tCBNDASPO** are increased. Nevertheless, its DF increase ratio is markedly smaller than that of DBFDPO:7% **tCBNDA**. Since PLQY values of **tCBNDASPO**-based films are always larger than those of **tCBNDA**-based analogs (Table S2), the shorter lifetimes of the former should be ascribed to faster singlet radiation and more efficient RISC.

Rate constants (*k*) and efficiencies (*ϕ*) of key TADF transitions were estimated to figure out effects of PO substitution on electronic characteristics of MR molecules (Figure 2(d) and Table S2). It is showed that ratio of PF (*ϕ*_PF_) and DF (*ϕ*_DF_) efficiencies is ~2 : 1 for DBFDPO:20% **tCBNDASPO**, on the contrary to ~1 : 3 for DBFDPO:7% **tCBNDA**. Especially, in accord with their *f*_*S*_ values (insets of Figure 2(d)), singlet radiative rate constant (*k*_*r*_^*S*^) of DBFDPO:20% **tCBNDASPO** reaches to 1.4 × 10^8^ s^−1^, which is more than 17 folds of that of DBFDPO:7% **tCBNDA**. Although rate constant of intersystem crossing (ISC) (*k*_ISC_) for DBFDPO:20% **tCBNDASPO** is twice of that of DBFDPO:7% **tCBNDA**, and RISC rate constant (*k*_RISC_) of the former is a half of the latter, the dynamic predominance of ISC for these two films is the same. However, it is noted that *k*_*r*_^*S*^/*k*_ISC_ ratio of DBFDPO:20% **tCBNDASPO** is 2 as ~8 folds of that of DBFDPO:7% **tCBNDA** (0.27). In addition, despite their comparable RISC quantum efficiencies (*ϕ*_RISC_) of ~95%, *η*_RISC_/ISC quantum efficiency (*ϕ*_ISC_) ratio of the former is more than 3 (*ϕ*_ISC_ = 31%), owing to overwhelming advantage of its *k*_*r*_^*S*^ to *k*_ISC_, which can largely reduce ISC-RISC cycles [51]. On the contrary, *ϕ*_RISC_/*ϕ*_ISC_ ratio of the latter is only 1.3. More efficient triplet-to-singlet conversion further reduces triplet quenching of DBFDPO:20% **tCBNDASPO**, whose triplet nonradiative rate constant (*k*_*nr*_^*T*^) is only one-third of that of DBFDPO:7% **tCBNDA**. Therefore, compared to **tCBNDA**, **tCBNDASPO** makes its film remarkably superior in singlet radiation and triplet harvesting.

### 2.3. Electroluminescence Performance

**Figure 3:**
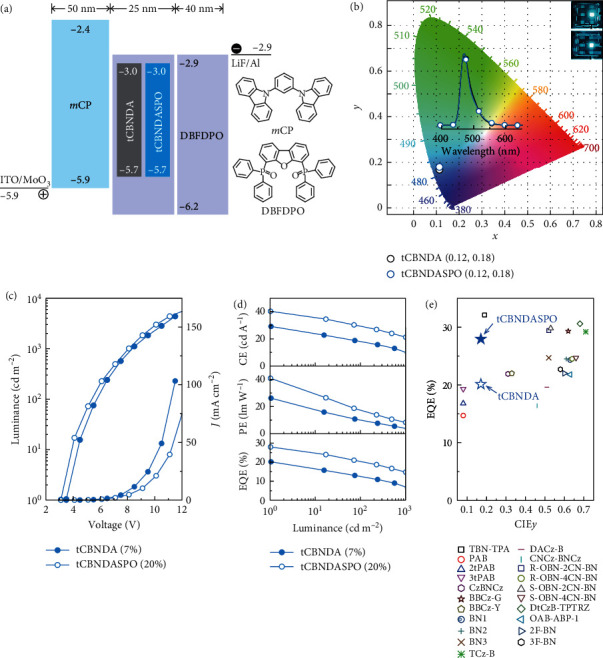
Electroluminescence (EL) performance of **tCBNDA** and **tCBNDASPO**. (a) Energy level diagram of simple trilayer device architecture based on a tri-material design, in which *m*CP is hole-transporting layer, and DBFDPO is used as both host and electron-transporting layer. (b) CIE 1931 chromaticity coordinates, corresponding EL spectra, and photos at 1000 nits of the blue devices. (c) Current density (*J*)-voltage (*V*)-luminescence (*L*) curves of **tCBNDA**- and **tCBNDASPO**-based OLEDs with the optimal doping concentrations of 7% and 20%, respectively. (d) Efficiencies-luminance curves of the optimized devices. (e) EQE comparison of all reported MR-TADF emitters modified with functional substituents on MR cores.

*η*_PL_ over 90%, bipolar modification and good film formability (Figure S19) of **tCBNDASPO** make device structural simplification feasible. Therefore, a four-material-based simple trilayer structure of ITO|MoO_3_ (6 nm)|*m*CP (50 nm)|DBFDPO:*x*% MR emitters (25 nm)|DBFDPO (40 nm)|LiF (1 nm)|Al was adopted to fabricate OLEDs through *vacuum* evaporation, in which *m*CP is 1,3-bis(9H-carbazol-9-yl)benzene as hole transporting layers, and DBFDPO simultaneously serves as host in emissive layer (EML) and electron-transporting layer (Figure 3(a)). The doping concentration (*x*%) was tuned to achieve the optimal device performance (Figures S20 and S21). All the devices revealed narrowband blue emissions peaked at 472 nm, corresponding to Commission Internationale de l'Eclairage (CIE) coordinates of 0.12 and 0.17-0.23 (Table S3). In accord with optical results, increasing *x*% induced electroluminescence (EL) red shift and FWHM increase by 4 nm for **tCBNDA**-based devices, respectively, corresponding to a maximum CIEy increase of 0.06. On the contrary, **tCBNDASPO**-based devices displayed unchanged EL peak wavelengths and FWHM values of 32 nm, corresponding to a negligible CIEy variation within 0.01. At *x* = 7 and 20, emission color purities of **tCBNDA**- and **tCBNDASPO**-based blue OLEDs were almost the same (Figure 3(b)).

The dependence of volt-ampere characteristics on *x*% was not distinct, revealing the predominance of DBFDPO host in carrier transportation of EMLs (Figures S17 and S21 and Table S1). However, at the same voltages, current densities (*J*) of **tCBNDASPO**-based devices were lower than those of **tCBNDA**-based analogs, while luminance of the former was higher than that of the latter. It means DPPO substitution improved carrier flux balance and recombination. Simultaneously, **tCBNDA**-based devices achieved the best performance at relative low *x*, e.g., the highest efficiencies of 29.3 cd A^−1^ for current efficiency (CE, *η*_CE_) and 27.0 lm W^−1^ for power efficiency (PE, *η*_PE_) at *x* = 10 and 20.2% for *η*_EQE_ at *x* = 7 (Figure 3(c) and S20 and Table S1). It indicates self-aggregation of **tCBNDA** worsens concentration quenching in its devices, which further increased roll-offs. In contrast, **tCBNDASPO**-based devices achieved the best performance at markedly higher *x*% (Figure S21). At *x* = 20, **tCBNDASPO** endowed its device with the state-of-the-art maximum efficiencies of 40.4 cd A^−1^, 40.9 lm W^−1^, and 28.0%, which were largely improved by 40%-50% in comparison to the best results of **tCBNDA**. At 100 nits, EL efficiencies of DBFDPO:20% **tCBNDASPO** still remained 29.8 cd A^−1^ and 20.6%, which were still beyond the highest values of **tCBNDA**-based devices. Obviously, the advantage of **tCBNDASPO** in more efficient RISC and triplet quenching reduction effectively alleviated triplet quenching, leading to markedly reduced EQE roll-offs.

EL performances of representative functionalized MR-TADF emitters are summarized in Figure 3(e) and Table S4. It is showed that EL emissions from most of them shifted to green, yellow, or orange with increased CIE*y* > 0.2. In comparison, **tCBNDASPO** realizes the combination of CIE*y* preservation and *η*_EQE_ improvement, reaching the top-rank levels of MR-TADF emitters.

**Figure 4:**
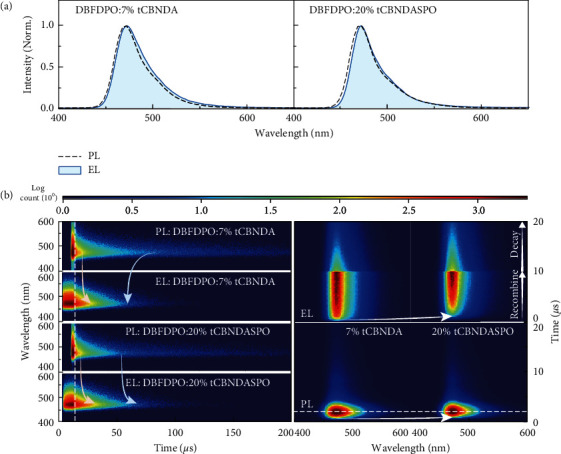
EL kinetics of **tCBNDA**- and **tCBNDASPO**-based OLEDs. (a) Comparison on PL and EL spectra of DBFDPO:7% **tCBNDA** and DBFDPO:20% **tCBNDASPO**. (b) PL and EL transient emission contours of DBFDPO:7% **tCBNDA** and DBFDPO:20% **tCBNDASPO** (left) and exciton formation in PL and EL processes.

Steady-state EL spectra of **tCBNDA** and **tCBNDASPO** were similar to PL spectra of their films, except for slight red shifts (Figure 4(a)). However, comparison on time-resolved PL and EL spectra indicates that DF component of DBFDPO:7% **tCBNDA** was predominant in PL process. But, in EL process, it was significantly reduced, at the same time of remarkable PF increasing (Figure 4(b)). On the contrary, PF and DF components of EL emission from DBFDPO:20% **tCBNDASPO** were simultaneously enhanced. It is showed that *J* of the devices was roughly in direct proportion to the doping concentrations of **tCBNDA** and **tCBNDASPO** (Figures S20 and S21). Therefore, due to the deeper LUMOs and shallower HOMOs of these two MR emitters than those of DBFDPO, direct carrier capture and recombination would be dominant in EL mechanism. Different to spin-forbidden triplet photo-excitation, electro-generated triplet excitons are formed directly through carrier recombination. Therefore, triplet concentration in devices follows spin statistics, which is larger than photo-excited triplets. Higher *k*_*nr*_^*T*^ and multiple ISC-RISC cycles of **tCBNDA** induced more serious triplet quenching and decreased EL DF components. For the same reason, owing to its halved *k*_*nr*_^*T*^, overwhelming thermodynamic advantage of RISC, and extremely high *k*_*r*_^*S*^ at the level of 10^8^ s^−1^, **tCBNDASPO** dramatically improved triplet harvesting in its devices, indicating the importance of functionalization for exciton utilization enhancement.

## 3. Discussion

In summary, a DPPO-modified blue MR emitter named **tCBNDASPO** is developed to demonstrate a feasible strategy for selectively improving TADF performance without scarifying narrowband emission feature. The insulation and induction effects of the P=O group are combined to, respectively, confine the *S*_1_ state on MR core and enhance key TADF transitions. Therefore, **tCBNDASPO** achieves preserved FWHM values (28 nm in film and 32 nm in device), 30% increased *ϕ*_PL_ (> 90%), 17-fold increased *k*_*r*_^*S*^ (10^8^ s^−1^), halved *k*_*nr*_^*T*^, and doubled *ϕ*_RISC_/*ϕ*_ISC_ ratio. Based on a trilayer simple structure, **tCBNDASPO** endowed its blue OLEDs with desired high color purity and the state-of-the-art *η*_EQE_ up to 28.0%. Therefore, linkage between MR frameworks and functional groups is crucial for selective optimization and purposeful system extension of MR-TADF materials.

## 4. Materials and Methods

Additional synthesis, Gaussian simulation results, electrochemical, photophysical and morphological properties, device performance, and NMR spectra are included in the Supplementary Materials.

## Data Availability

All other data are available from the authors upon reasonable request.

## Supplementary Materials

Supplementary Materials Scheme S1: synthetic procedure of **tCBNDASPO**: (i) 3,6-tert-butylcarbazole, K2CO3, CuI, 18-crown-6, DMI, 190°C, 48 h; (ii) diphenylamine, t-BuOK, Pd2dba3, tri(tert-butyl)phosphine, toluene, 90°C, 5 h; (iii) BBr3, toluene, 120°C, 20 h; (iv) NBS, CH2Cl2, r.t., 30 min; (v) diphenylphosphine, Pd(OAc)2, NaOAc, DMF, 130°C, 24 h; 30% H2O2, CH2Cl2, 0°C, 1 h. Figure S1: contours and energy levels of the first three frontier molecular orbitals of monomer of **tCBNDA** and **tCBNDASPO** simulated with the DFT method. Figure S2: contours of “hole” and “particle” of *S*_0_⟶*S*_1_ and *S*_0_⟶*T*_1_ excitations for **tCBNDA** simulated with the TD-DFT method. *E*_*S*_, *E*_*T*_, *f*, and *σ* refer to the energy levels of the *S*_1_ and the *T*_1_ states, singlet oscillator strength, and contribution weight. Figure S3: contours of “hole” and “particle” of *S*_0_⟶*S*_1_ and *S*_0_⟶*T*_1_ excitations for **tCBNDASPO** simulated with the TD-DFT method. *E*_*S*_, *E*_*T*_, *f*, and *σ* refer to the energy levels of the *S*_1_ and the *T*_1_ states, singlet oscillator strength, and contribution weight. Figure S4: centroid-centroid distances of FMOs (dH-L) and overlap integrals of FMO wave functions (〈Ψ_*H*_|Ψ_*L*_〉) and electron cloud densities (〈Ψ_*H*_^2^|Ψ_*L*_^2^〉) of **tCBNDA** and **tCBNDASPO** at the *S*_0_, *S*_1_, and the *T*_1_ states. Figure S5: contours of “hole” and “particle” of *S*_0_⟶*S*_2_ and *S*_0_⟶*T*_2_ excitations for **tCBNDA** simulated with the TD-DFT method. *E*_*S*_, *E*_*T*_, *f*, and *σ* refer to the energy levels of the *S*_2_ and the *T*_2_ states, singlet oscillator strength, and contribution weight. Figure S6: contours of “hole” and “particle” of *S*_0_⟶*S*_2_ and *S*_0_⟶*T*_2_ excitations for **tCBNDASPO** simulated with the TD-DFT method. *E*_*S*_, *E*_*T*_, *f*, and *σ* refer to the energy levels of the *S*_2_ and the *T*_2_ states, singlet oscillator strength, and contribution weight. Figure S7: Illustration of DF processes for **tCBNDA** and **tCBNDASPO**. VC and NR refer to vibrational coupling and nonradiation. Figure S8: cyclic voltammogram of **tCBNDA** and **tCBNDASPO** measured in DCM for oxidation and THF for reduction at room temperature with the scanning rate of 100 mV s-1 and tetra-n-butylammonium hexafluorophosphate as supporting electrolyte (0.1 mol L-1). Figure S9: electronic absorption and photoluminescence (PL) spectra of (a) **tCBNDA** and (b) **tCBNDASPO** in different solvents with diverse polarities. Figure S10: variation of PL spectra for DBFDPO:*x*% **tCBNDA** and DBFDPO:*x*% **tCBNDASPO** films. Figure S11: dependence of FWHM and PLQY values for DBFDPO:*x*% **tCBNDA** and DBFDPO:*x*% **tCBNDASPO** films on doping concentration *x*%. Figure S12: concentration dependence of PF (a) and DF (b) decay curves for DBFDPO:*x*% **tCBNDA** films. Figure S13: concentration dependence of PF (a) and DF (b) decay curves for DBFDPO:*x*% **tCBNDASPO** films. Figure S14: temperature dependence of DF decays for neat **tCBNDA** (a) and **tCBNDASPO** (b) films. Figure S15: temperature dependence of DF decays for DBFDPO:7% **tCBNDA** (a) and DBFDPO:20% **tCBNDASPO** (b) films. Figure S16: temperature dependence of time-resolved emission spectra (TRES) for neat **tCBNDA** (a) and **tCBNDASPO** (b) films. Figure S17: temperature dependence of time-resolved emission spectra (TRES) for DBFDPO:7% **tCBNDA** (a) and DBFDPO:20% **tCBNDASPO** (b) films. Figure S18: concentration dependence of time-resolved emission spectra (TRES) for DBFDPO:*x*% **tCBNDA** (a) and DBFDPO:*x*% **tCBNDASPO** (b) films. Figure S19: atom force microscopy (AFM) images of neat films and DBFDPO:7% **tCBNDA** and DBFDPO:20% **tCBNDASPO** films. Figure S20: (a) EL spectra (inset) and luminance-current density (*J*)-voltage curves of the devices with diffferent **tCBNDA** doping concentrations of 5%, 7%, 10%, and 20%; (b) efficiencies vs. luminance relationships of the devices. Figure S21: (a) EL spectra (inset) and luminance-current density (*J*)-voltage curves of the devices with diffferent **tCBNDASPO** doping concentrations of 10%, 20%, 30%, and 40%; (b) efficiencies vs. luminance relationships of the devices. Figure S22: ^1^H NMR spectrum of **tCBNDA** in CDCl_3_. Figure S23: ^1^H NMR spectrum of **tCBNDASPO** in CDCl_3_. Figure S24: ^13^C NMR spectrum of **tCBNDA** in CDCl_3_. Figure S25: ^13^C NMR spectrum of **tCBNDASPO** in CDCl_3_. Figure S26: ^31^PNMR spectrum of **tCBNDASPO** in CDCl_3_. Table S1: basic physical properties of **tCBNDA** and **tCBNDASPO**. Table S2: TADF characteristics of DBFDPO:*x*% **tCBNDA** and DBFDPO:*x*% **tCBNDASPO** films. Table S3: EL performance of DBFDPO:*x*% **tCBNDA** and DBFDPO:*x*% **tCBNDASPO**. Table S4: EL performance of representative functionalized MR-TADF emitters.
