# Supplementary material for: Synergetic Insulation and Induction Effects Selectively Optimize Multiresonance Thermally Activated Delayed Fluorescence
Source: Research (Wash D C). 2022 Jun 2;2022:9838120. doi: 10.34133/2022/9838120 (PMC9275084; doi:10.34133/2022/9838120)
Supplement: Supplementary Materials — Scheme S1: synthetic procedure of tCBNDASPO: (i) 3,6-tert-butylcarbazole, K2CO3, CuI, 18-crown-6, DMI, 190°C, 48 h; (ii) diphenylamine, t-BuOK, Pd2dba3, tri(tert-butyl)phosphine, toluene, 90°C, 5 h; (iii) BBr3, toluene, 120°C, 20 h; (iv) NBS, CH2Cl2, r.t., 30 min; (v) diphenylphosphine, Pd(OAc)2, NaOAc, DMF, 130°C, 24 h; 30% H2O2, CH2Cl2, 0°C, 1 h. Figure S1: contours and energy levels of the first three frontier molecular orbitals of monomer of tCBNDA and tCBNDASPO simulated with the DFT method. Figure S2: contours of “hole” and “particle” of S0⟶S1 and S0⟶T1 excitations for tCBNDA simulated with the TD-DFT method. ES, ET, f, and σ refer to the energy levels of the S1 and the T1 states, singlet oscillator strength, and contribution weight. Figure S3: contours of “hole” and “particle” of S0⟶S1 and S0⟶T1 excitations for tCBNDASPO simulated with the TD-DFT method. ES, ET, f, and σ refer to the energy levels of the S1 and the T1 states, singlet oscillator strength, and contribution weight. Figure S4: centroid-centroid distances of FMOs (dH-L) and overlap integrals of FMO wave functions (〈ΨH|ΨL〉) and electron cloud densities (〈ΨH2|ΨL2〉) of tCBNDA and tCBNDASPO at the S0, S1, and the T1 states. Figure S5: contours of “hole” and “particle” of S0⟶S2 and S0⟶T2 excitations for tCBNDA simulated with the TD-DFT method. ES, ET, f, and σ refer to the energy levels of the S2 and the T2 states, singlet oscillator strength, and contribution weight. Figure S6: contours of “hole” and “particle” of S0⟶S2 and S0⟶T2 excitations for tCBNDASPO simulated with the TD-DFT method. ES, ET, f, and σ refer to the energy levels of the S2 and the T2 states, singlet oscillator strength, and contribution weight. Figure S7: Illustration of DF processes for tCBNDA and tCBNDASPO. VC and NR refer to vibrational coupling and nonradiation. Figure S8: cyclic voltammogram of tCBNDA and tCBNDASPO measured in DCM for oxidation and THF for reduction at room temperature with the scanning rate of 100 mV s-1 and t [file 9838120.f1.docx]

**Supplementary Information**

**Synergetic Insulation and Induction Effects Selectively Optimize Multi-Resonance Thermally Activated Delayed Fluorescence**

*Jinkun Bian, Lili Qiu, Su Chen,* *Nan Zhang, Jing Zhang, Chunbo Duan, Chunmiao Han, and Hui Xu**

Key Laboratory of Functional Inorganic Material Chemistry, Ministry of Education & School of Chemistry and Material Science, Heilongjiang University, Harbin, Heilongjiang 150080, China.

**Content**

[I. Experimental Section 2](#_Toc88164372)

[II. Gaussian simulation results 9](#_Toc88164373)

[III. Electrochemical properties 12](#_Toc88164374)

[IV. Photophysical properties 13](#_Toc88164375)

[V. Morphological properties 22](#_Toc88164376)

[VI. OLED performances 23](#_Toc88164377)

[Table S1. Basic physical properties of tCBNDA and tCBNDASPO. 25](#_Toc88164378)

[Table S2. TADF characteristics of DBFDPO:*x*% tCBNDA and DBFDPO:*x*% tCBNDASPO films. 26](#_Toc88164379)

[Table S3. EL performance of DBFDPO:*x*% tCBNDA and DBFDPO:*x*% tCBNDASPO. 27](#_Toc88164380)

[Table S4. EL performance of representative functionalized MR-TADF emitters. 28](#_Toc88164381)

[VII. References 35](#_Toc88164382)

### I. Experimental Section

**1. Materials and Instruments**

All the reagents and solvents were purchased from Aldrich and Acros companies and used without further purification.

^1^H NMR spectra were recorded using a Varian Mercury plus 400NB spectrometer relative to tetramethylsilane (TMS) as internal standard. Molecular masses were determined by a FINNIGAN LCQ Electro-Spraying Ionization-Mass Spectrometry (ESI-MS), or a MALDI-TOF-MS. Elemental analyses were performed on a Vario EL III elemental analyzer. Absorption and photoluminescence (PL) emission spectra of the target compound were measured using a SHIMADZU UV-3150 spectrophotometer and a SHIMADZU RF-5301PC spectrophotometer, respectively. Thermogravimetric analysis (TGA) and differential scanning calorimetry (DSC) were performed on Shimadzu DSC-60A and DTG-60A thermal analyzers under nitrogen atmosphere at a heating rate of 10 °C min^-1^. Cyclic voltammetric (CV) studies were conducted using an Eco Chemie B. V. AUTOLAB potentiostat in a typical three-electrode cell with a platinum sheet working electrode, a platinum wire counter electrode, and a silver/silver nitrate (Ag/Ag^+^) reference electrode. All electrochemical experiments were carried out under a nitrogen atmosphere at room temperature in dichloromethane. Phosphorescence spectra were measured in dichloromethane using an Edinburgh FPLS 1000 fluorescence spectrophotometer at 77 K cooling by liquid nitrogen. The time decay spectra was measured using Time-Correlated Single Photon Counting (TCSPC) method with a picosecond hydrogen lamp for 100 *p*s-10 *μ*s and a microsecond pulsed Xenon light source for 1 *μ*s-10 s lifetime measurement, the synchronization photomultiplier for signal collection and the Multi-Channel Scaling Mode of the PCS900 fast counter PC plug-in card for data processing. Lifetime values were simulated by single exponential fitting function in Fluoracle software.

**2. Synthesis**

**Scheme S1.** Synthetic procedure of **tCBNDASPO**: i. 3,6-*tert*-butylcarbazole, K_2_CO_3_, CuI, 18-crown-6, DMI, 190 ℃, 48 h; ii. Diphenylamine, *t*-BuOK, Pd_2_dba_3_, tri(*tert*-butyl)phosphine, toluene, 90 ℃, 5 h; iii. BBr_3_, toluene, 120 ^o^C, 20 h; iv. NBS, CH_2_Cl_2_, r.t., 30 min; v. diphenylphosphine, Pd(OAc)_2_, NaOAc, DMF, 130 ^o^C, 24 h; 30% H_2_O_2_, CH_2_Cl_2_, 0 ^o^C, 1 h.

**3,6-Di-*tert*-butyl-9-(3,5-dibromophenyl)-9H-carbazole (1)**

In Ar, a mixture of 1.3.5-tribromobenzene (3.14 g, 10 mmol), tert-butylcarbazole (2.79 g, 10 mmol), 18-crown-6 (0.132 g, 0.5 mmol), CuI (0.19 g 1 mmol) and potassium carbonate (2.76 g, 20 mmol) in 10 mL of DMI was stirred and heated to 190 ^o^C for 48 hours. The mixture was then cooled to room temperature, and extracted with water and CH_2_Cl_2_ (3 × 30 mL). The organic layer was combined and dried with anhydrous Na_2_SO_4_. The solvent was removed in vacuo. The residue was purified by flash column chromatography with PE as eluent to afford white powder of 3.7 g with a yield of 72.4%. ^1^H NMR (TMS, CDCl_3_, 400 MHz): *δ* = 8.118 (s, 2H), 7.710-7.685 (m, 3H), 7.498-7.472 (m, 2H), 7.369-7.348 (d, *J* = 8.8 Hz, 2H), 1.460 ppm (s, 18H); ^13^C NMR (TMS, CDCl_3_, 100 MHz): *δ* = 143.834, 138.535, 132.387, 128.288, 124.000, 123.807, 123.666, 116.480, 109.029, 77.373, 77.056, 76.738, 34.817, 31.998 ppm; LDI-TOF: m/z (%) 513 (100) [M^+^]; Elemental Analysis for C_26_H_27_Br_2_N: C 60.84, H 5.30, N 2.73; found: C 60.85, H 5.29, N 2.75.

**5-(3,6-Di-tert-butyl-9H-carbazol-9-yl)-*N1*,*N1*,*N3*,*N3*-tetraphenylbenzene-1,3-diamine (2)**

In Ar, a mixture of **1** (11.24 g, 22 mmol), tert-butylcarbazole (2.79 g, 10 mmol), diphenylamine (8.1 g, 48 mmol) and sodium tert-butoxide (6.38 g, 66 mmol) in 75 mL of toluene was stirred at room temperature for 30 min. Then, tris-(dibenzylideneacetone)dipalladium(0) (0.304 g, 0.332 mmol) and tri-*tert*-butyl phosphine (0.32 mL, 0.775 mmol, 50% in toluene) were added. The mixture was stirred at 90 ^o^C for 5 hours, and then cooled to room temperature, and extracted with water and CH_2_Cl_2_ (3 × 30 mL). The organic layer was combined and dried with anhydrous Na_2_SO_4_. The solvent was removed in vacuo. The residue was purified by flash column chromatography with PE:DCM (8:1) as eluent to afford white powder of 10.2 g with a yield of 67.3%. ^1^H NMR (TMS, CDCl_3_, 400 MHz): *δ* = 8.044-8.040 (d, *J* = 1.2 Hz, 2H), 7.408-7.383 (m, 2H), 7.336-7.314 (d, *J* = 8.8 Hz, 2H), 7.235-7.215 (d, *J* = 8 Hz, 8H), 7.156-7.136 (d, *J* = 8 Hz, 8H), 7.001-6.965 (t, *J* = 7.2 Hz, 4H), 6.838-6.809 (m, 3H), 1.428 ppm (s, 18H); ^13^C NMR (TMS, CDCl_3_, 100 MHz): *δ* = 149.607, 147.202, 142.723, 139.539, 139.634, 129.284, 124.437, 123.389, 123.342, 123.214, 116.408 ,118.183, 115.062, 109.430, 77.378, 77.060, 76.743, 58.508, 34.708, 32.091, 18.461 ppm; LDI-TOF: m/z (%) 689 (100) [M^+^]; Elemental Analysis for C_50_H_47_N_3_: C 87.04, H 6.87, N 6.09; found: C 87.06, H 6.85, N 6.09.

**11,14-Di-tert-butyl-N,N,5-triphenyl-5H-5,8b-diaza-15b-borabenzo[a]naphtho[1,2,3-hi]aceanthrylen-7-amine (tCBNDA)**

In Ar, **2** (0.689 g, 1 mmol) was dissolved in 10 mL of toluene, and then boron tribromide (1.14 mL, 12 mmol) was added. Then, the mixture was stirred at 120 ^o^C for 20 hours, and then cooled to room temperature. After adding phosphorus buffer solution (pH = 7, 20 mL), the aqueous layer was extracted with CH_2_Cl_2_ (3 × 30 mL). The organic layer was combined and dried with anhydrous Na_2_SO_4_. The solvent was removed in vacuo. The residue was purified by flash column chromatography with PE:DCM (10:1) to provide green powder of 0.2 g with a yield of 28.6%. ^1^H NMR (TMS, CDCl_3_, 400 MHz): *δ* = 8.990-8.971 (d, *J* = 7.6 Hz, 1H), 8.941 (s, 1H), 8.352 (s, 1H), 8.181 (s, 1H), 7.617-7.593 (d, *J* = 9.6 Hz, 2H), 7.510-7.472 (t, *J* = 7.6 Hz, 2H), 7.432-7.368 (m, 2H), 7.324-7.192 (m, 12H), 7.126-7.090 (t, *J* = 7.2 Hz, 2H), 6.751-6.730 (d, *J* = 8.4 Hz, 2H), 5.864 (s, 1H), 1.631 (s, 9H), 1.463 ppm (s, 9H); ^13^C NMR (TMS, CDCl_3_, 100 MHz): *δ* = 149.738, 147.158, 140.299, 139.053, 129.321, 125.763, 124.612, 123.381, 123.351, 120.198, 119.833 ,116.124, 114.930, 110.054, 77.368, 77.050, 76.733 ppm; LDI-TOF: m/z (%) 697 (100) [M^+^]; Elemental Analysis for C_50_H_44_BN_3_: C 86.07, H 6.36, N 6.02; found: C 86.08, H 6.35, N 6.04.

***N*-(4-bromophenyl)-11,14-di-tert-butyl-*N*,5-diphenyl-5H-5,8b-diaza-15b-borabenzo[a]naphtho[1,2,3-hi]aceanthrylen-7-amine (tCBNDABr)**

In Ar, **tCBNDA** (3.10 g, 4 mmol) was dissolved in 20 mL of dichloromethane, and then *N*-bromosuccinimide (NBS, 0.708g, 4 mmol) was added in proportion. The mixture was stirred at room temperature for 30 min. The mixture was then extracted with water and CH_2_Cl_2_ (3 × 30 mL). The organic layer was combined and dried with anhydrous Na_2_SO_4_. The solvent was removed in vacuo. The residue was purified by flash column chromatography with PE:DCM (12:1) to provide green powder of 2.1 g with a yield of 67.7%. ^1^H NMR (TMS, CDCl_3_, 400 MHz): *δ* = 8.994-8.975 (d, *J* = 7.6Hz, 1H), 8.946 (s, 1H), 8.365 (s, 1H), 8.194 (s, 1H), 7.631-7.613 (m, 2H), 7.538-7.501 (t, *J* = 7.6 Hz, 2H), 7.455-7.405 (m, 2H), 7.371-7.279 (m, 7H), 7.227-7.186 (m, 3H), 7.160-7.124 (t, *J* = 7.2 Hz, 2H), 7.053-7.031 (d, *J* = 8.8 Hz, 2H), 6.774-6.752 (d, *J* = 8.8 Hz, 1H), 5.802 (s, 1H), 1.633 (s, 9H), 1.471 ppm (s, 9H); ^13^C NMR (TMS, CDCl_3_, 100 MHz): *δ* = 151.381, 148.782, 147.342, 146.520, 146.120, 144.964, 144.508, 143.671, 141.964, 141.933, 137.820, 135.336, 132.277 ,131.143, 130.823, 130.128, 129.501, 128.454, 127.172 , 126.943, 125.981, 124.504, 123.974, 123.292, 120.186, 119.393, 117.087, 117.068, 116.551, 113.284, 101.363, 99.140, 77.375, 77.057, 76.739, 35.212, 34.754, 32.324, 31.850 ppm; LDI-TOF: m/z (%) 775 (100) [M^+^]; Elemental Analysis for C_50_H_43_BBrN_3_: C 77.33, H 5.58, N 5.41; found: C 77.35, H 5.59, N 5.44.

**(4-((11,14-di-tert-butyl-5-phenyl-5H-5,8b-diaza-15b-borabenzo[a]naphtho[1,2,3-hi]aceanthrylen-7-yl)(phenyl)amino)phenyl)diphenylphosphine oxide (tCBNDASPO)**

In Ar, **tCBNDABr** (1.54g, 2 mmol), diphenylphosphine (0.32 ml, 2 mmol), palladium acetate (0.0179 g, 0.08 mmol) and sodium acetate (0.164 g 2 mmol) were dissolved in 10 mL of DMF, heated to 130 ^o^C and stirred for 24 hours. After cooling to room The dichloromethane layer was concentrated to 30 mL, and then 30% H_2_O_2_ (0.3 ml, 2 mmol) was added at 0 ^o^C. The mixture was then stirred for 1 hours, and then washed with sodium bisulfite aqueous solution. The mixture was extracted from water and dichloromethane (3 × 30 mL) again. The organic layer was combined and dried with anhydrous Na_2_SO_4_. The solvent was removed in vacuo. The residue was purified by flash column chromatography with EA:PE (1:2) to provide green powder of 0.98 g with a yield of 54.7%. ^1^H NMR (TMS, CDCl_3_, 400 MHz): *δ* = 8.994-8.976 (d, *J* = 7.2Hz, 1H), 8.944 (s, 1H), 8.369 (s, 1H), 8.191 (s, 1H), 7.736-7.661 (m, 5H), 7.606-7.557 (m, 3H), 7.526-7.414 (m, 10H), 7.364-7.286 (m, 5H), 7.229-7.192 (m, 2H), 6.761-6.739 (d, *J* = 8.8 Hz, 2H), 5.997 (s, 1H) , 1.632 (s, 9H), 1.463 ppm (s, 9H); ^13^C NMR (TMS, CDCl_3_, 100 MHz): *δ* = 150.646, 150.348, 148.706, 147.328, 146.245, 145.007, 144.642, 143.710, 141.938, 141.885, 137.795 ,135.368, 133.519, 133.151, 133.043, 132.480, 132.169 , 132.070, 131.945, 131.921, 131.267, 130.822, 130.117, 129.754, 129.567, 128.589, 128.469, 127.153, 126.985, 125.422, 123.956, 123.355, 122.518, 122.392, 120.311, 119.528, 117.112, 113.247, 102.762, 100.614, 77.365, 77.048, 76.730, 34.753, 32.299, 31.841 ppm; ^31^P NMR (TMS, CDCl_3_, 162 MHz): *δ* = 29.552 ppm; LDI-TOF: m/z (%) 897 (100) [M^+^]; Elemental Analysis for C_62_H_53_BN_3_OP: C 82.94, H 5.95, N 4.68; found: C 82.96, H 5.94, N 4.70.

**3. DFT Calculations**

DFT computations were carried out with different parameters for structure optimizations and vibration analyses. The ground states of molecules in *vacuum* were optimized without any assistance of experimental data by the restricted and unrestricted formalism of Beck's three-parameter hybrid exchange functional(*1*) and Lee, and Yang and Parr correlation functional(*2*) (B3LYP) for C, H, N, O and P. The optimization was performed at the level of 6-31G(d). The fully optimized stationary points were further characterized by harmonic vibrational frequency analysis to ensure that real local minima had been found without imaginary vibrational frequency. The total energies were also corrected by zero-point energy both for the ground state. All computations were performed using the Gaussian 03 package.(*3*)

**4. Device Fabrication and Testing**

Before loading into a deposition chamber, the ITO substrate was cleaned with detergents and deionized water, dried in an oven at 120 °C for 4 h, and treated with UV-ozone for 20 min. Devices were fabricated by evaporating organic layers at a rate of 0.1-0.3 nm s^-1^ onto the ITO substrate sequentially at a pressure below 1×10^-6^ mbar. Onto the electron transporting layer, a layer of LiF with 1 nm thickness was deposited at a rate of 0.1 nm s^-1^ to improve electron injection. Finally, a 100-nm-thick layer of Al was deposited at a rate of 0.6 nm s^-1^ as the cathode. The emission area of the devices was 0.09 cm^2^ as determined by the overlap area of the anode and the cathode. The EL spectra and CIE coordinates were measured using a PR655 spectra colorimeter. The current-density-voltage and brightness-voltage curves of the devices were measured using a Keithley 4200 source meter and a calibrated silicon photodiode. All the measurements were carried out at room temperature in glove box. EL transient spectra were measured with Edinburgh FLS1000 equipped with a Tektronix AFG3022G function generator. The voltage pulse amplitude was 5 V. The pulse width was 20 *μ*s.

**5. Calculation Formulas for the Photophysical Parameters**

The calculation formulas for the rate constants of prompt fluorescence (*k*_PF_) and delayed fluorescence (*k*_DF_), rate constant of singlet radiation ($k_{r}^{S}$), rate constants of singlet ($k_{nr}^{S}$) and triplet nonradiation ($k_{nr}^{T}$), quantum efficiencies of intersystem crossing (*η*_ISC_) and reverse intersystem crossing (*η*_RISC_) are expressed as following list:(*4, 5*)

$k_{\mathrm{PF}}=k_{r}^{S}+k_{nr}^{S}+k_{\mathrm{ISC}}$ (Eq. S1)

$k_{\mathrm{DF}}=k_{nr}^{T}+\left( 1-\frac{k_{\mathrm{ISC}}}{k_{\mathrm{PF}}} \right)\cdot k_{\mathrm{RISC}}$ (Eq. S2)

$k_{r}^{S}=\eta_{\mathrm{PF}}\cdot k_{\mathrm{PF}}$ (Eq. S3)

$k_{nr}^{S}=k_{\mathrm{PF}}-k_{r}^{S}-k_{\mathrm{ISC}}=k_{\mathrm{PF}}-k_{r}^{S}-{\left( 1-\eta_{PF} \right)\cdot k}_{\mathrm{PF}}$ (Eq. S4)

$k_{nr}^{T}=k_{\mathrm{DF}}-\left( 1-\frac{k_{\mathrm{ISC}}}{k_{\mathrm{PF}}} \right)\cdot k_{\mathrm{RISC}}=k_{\mathrm{DF}}-\left( 1-\frac{k_{\mathrm{ISC}}}{k_{\mathrm{PF}}} \right)\cdot\frac{k_{\mathrm{DF}}\cdot k_{\mathrm{PF}}\cdot\eta_{\mathrm{DF}}}{k_{\mathrm{ISC}}\cdot\eta_{\mathrm{PF}}}$ (Eq. S5)

$\eta_{\mathrm{ISC}}=\frac{k_{\mathrm{ISC}}}{k_{\mathrm{PF}}}$ (Eq. S6)

$\eta_{\mathrm{RISC}}=\frac{k_{\mathrm{RISC}}}{k_{\mathrm{RISC}}+k_{nr}^{T}}$ (Eq. S7)

### II. Gaussian simulation results


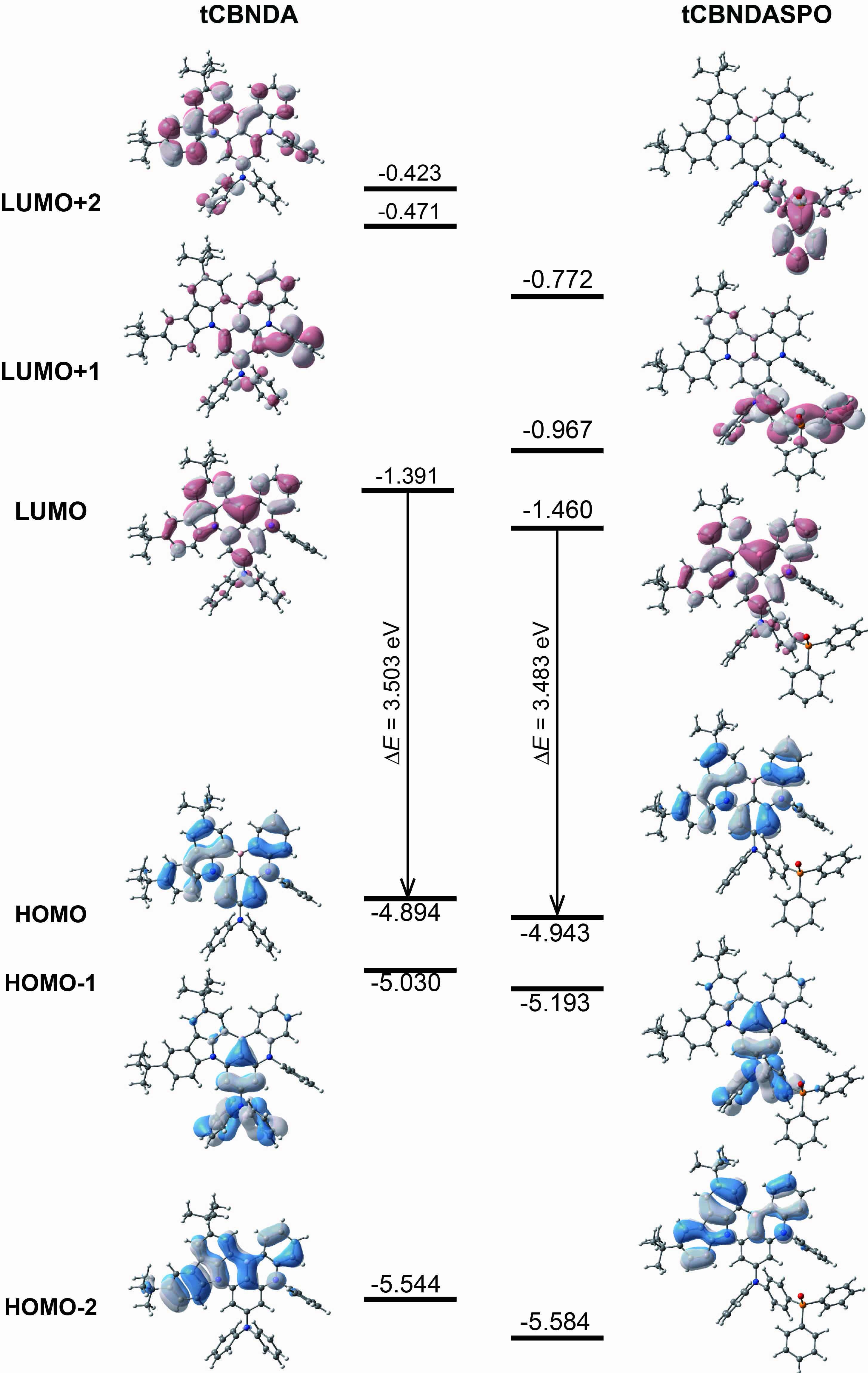


**Figure S1.** Contours and energy levels of the first three frontier molecular orbitals of monomer of **tCBNDA** and **tCBNDASPO** simulated with the DFT method.


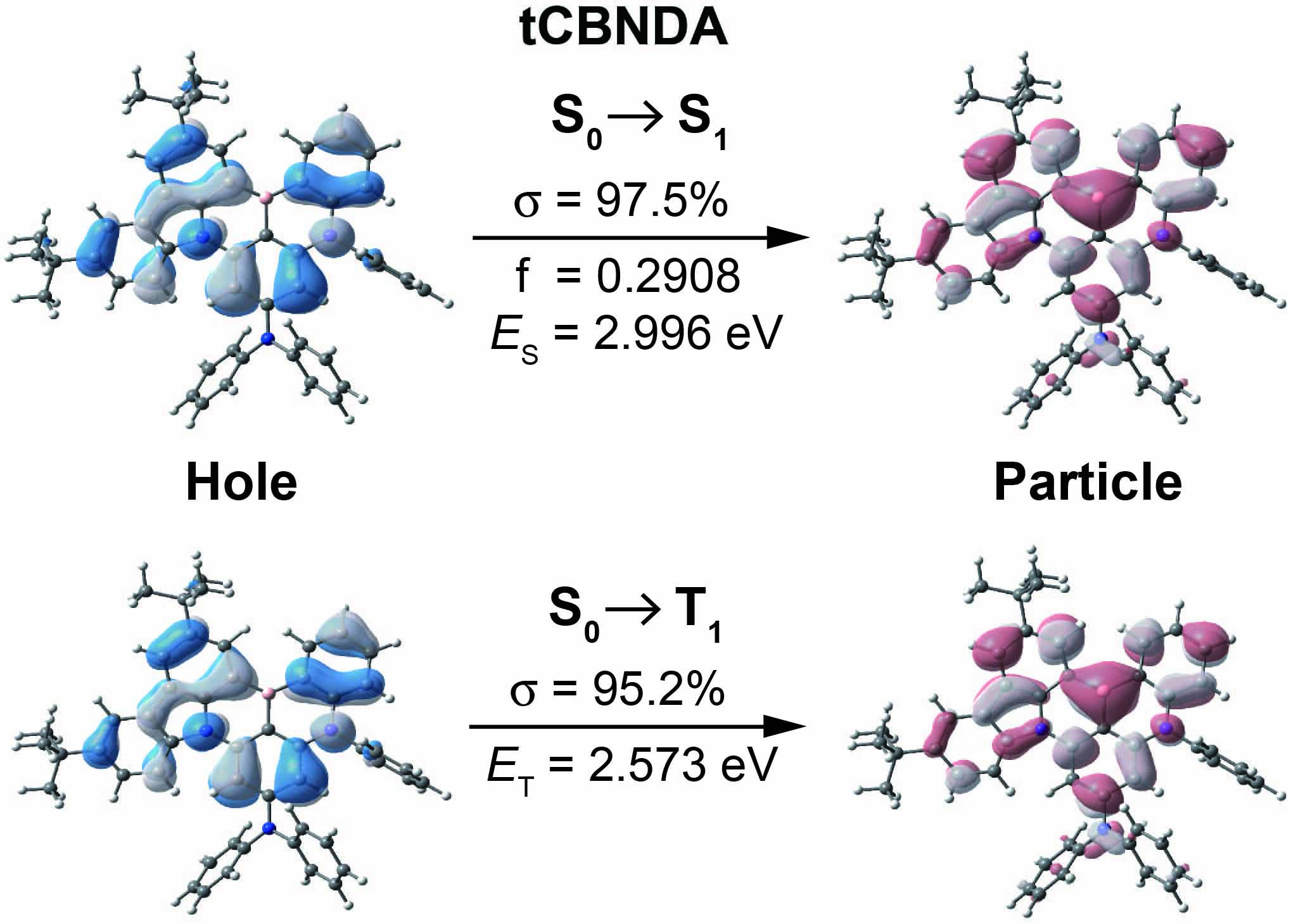


**Figure S2.** Contours of “hole” and “particle” of S_0_→S_1_ and S_0_→T_1_ excitations for **tCBNDA** simulated with the TD-DFT method. *E*_S_, *E*_T_, *f* and σ refer to the energy levels of the S_1_ and the T_1_ states, singlet oscillator strength and contribution weight.


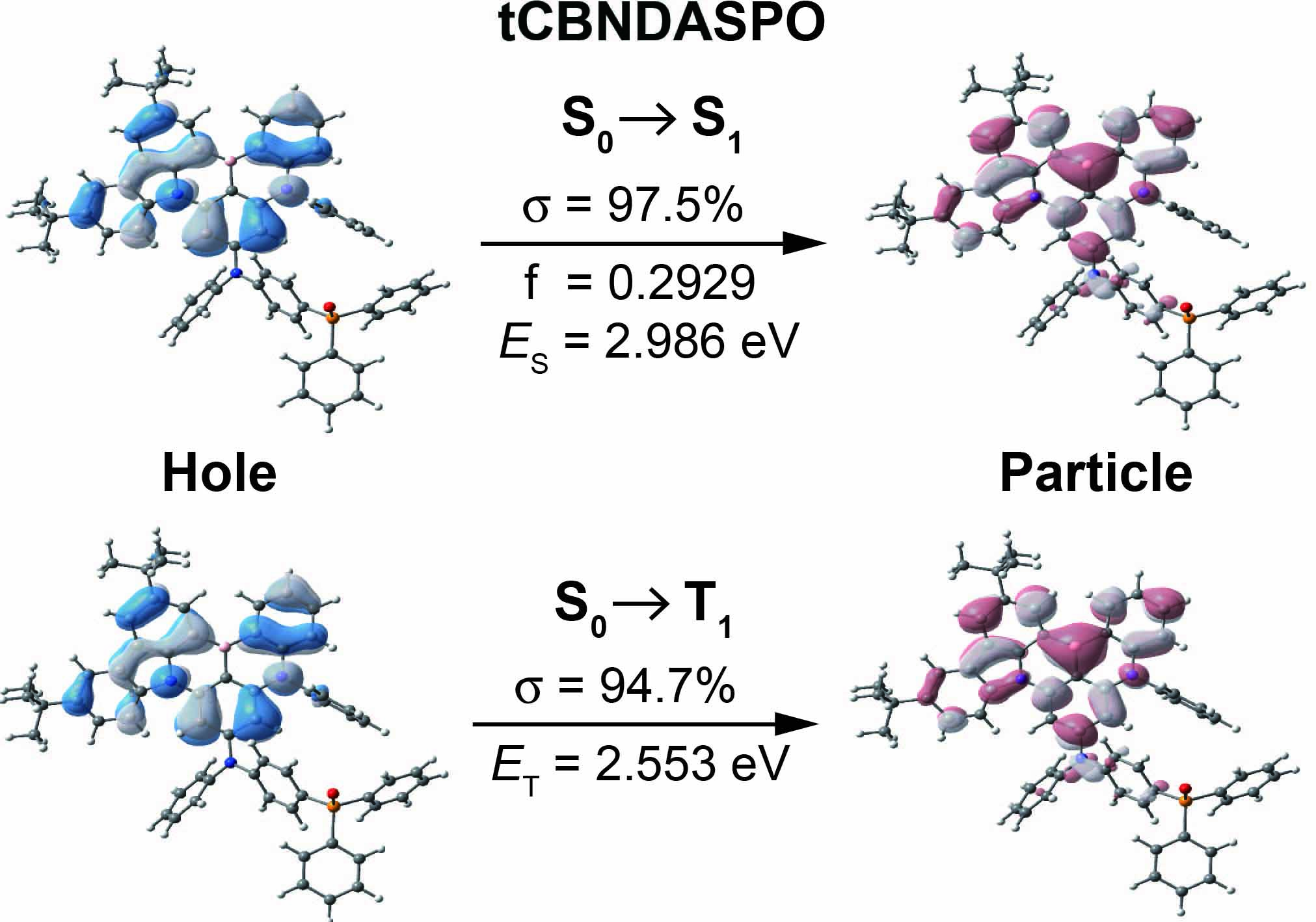


**Figure S3.** Contours of “hole” and “particle” of S_0_→S_1_ and S_0_→T_1_ excitations for **tCBNDASPO** simulated with the TD-DFT method. *E*_S_, *E*_T_, *f* and σ refer to the energy levels of the S_1_ and the T_1_ states, singlet oscillator strength and contribution weight.


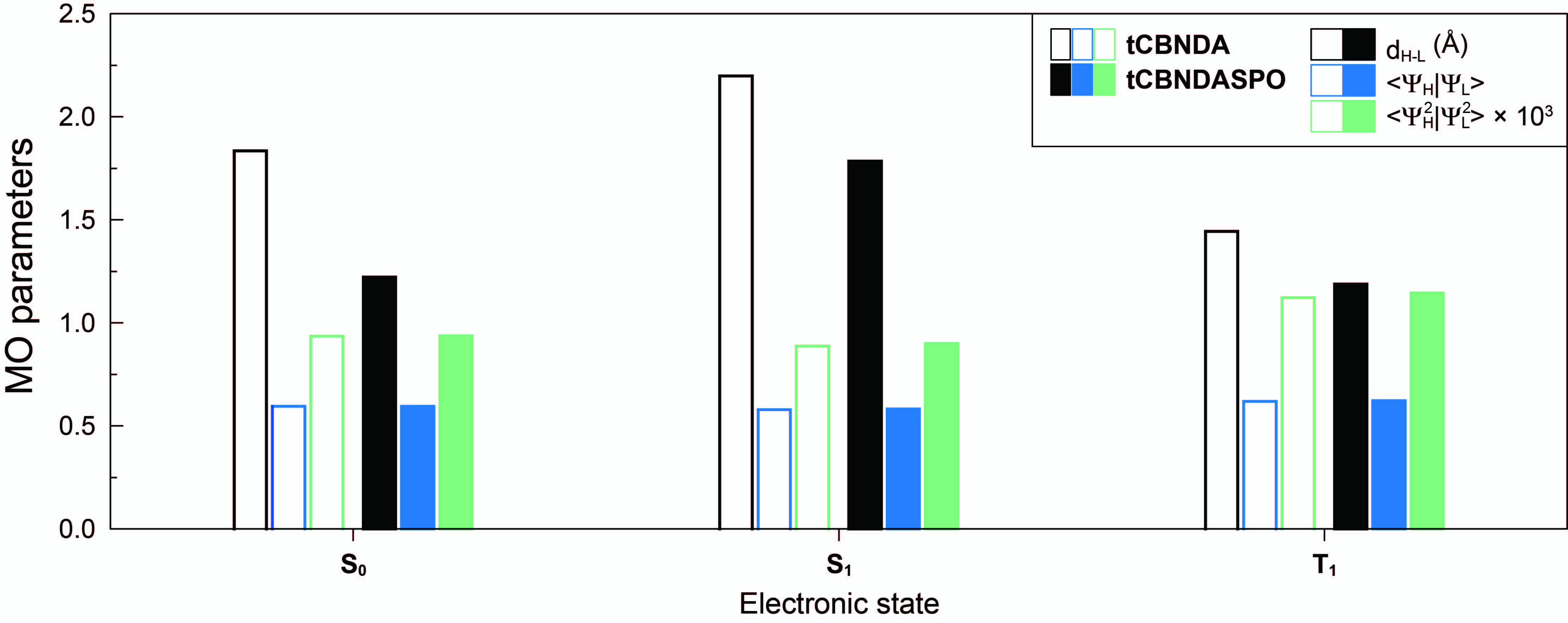


**Figure S4.** Centroid-centroid distances of FMOs (d*_H-L_*), and overlap integrals of FMO wave functions ($\left\langle\Psi_{H} | \Psi_{L} \right\rangle$) and electron cloud densities ($\left\langle\Psi_{H}^{2} | \Psi_{L}^{2} \right\rangle$) of **tCBNDA** and **tCBNDASPO** at the S_0_, S_1_ and the T_1_ states.


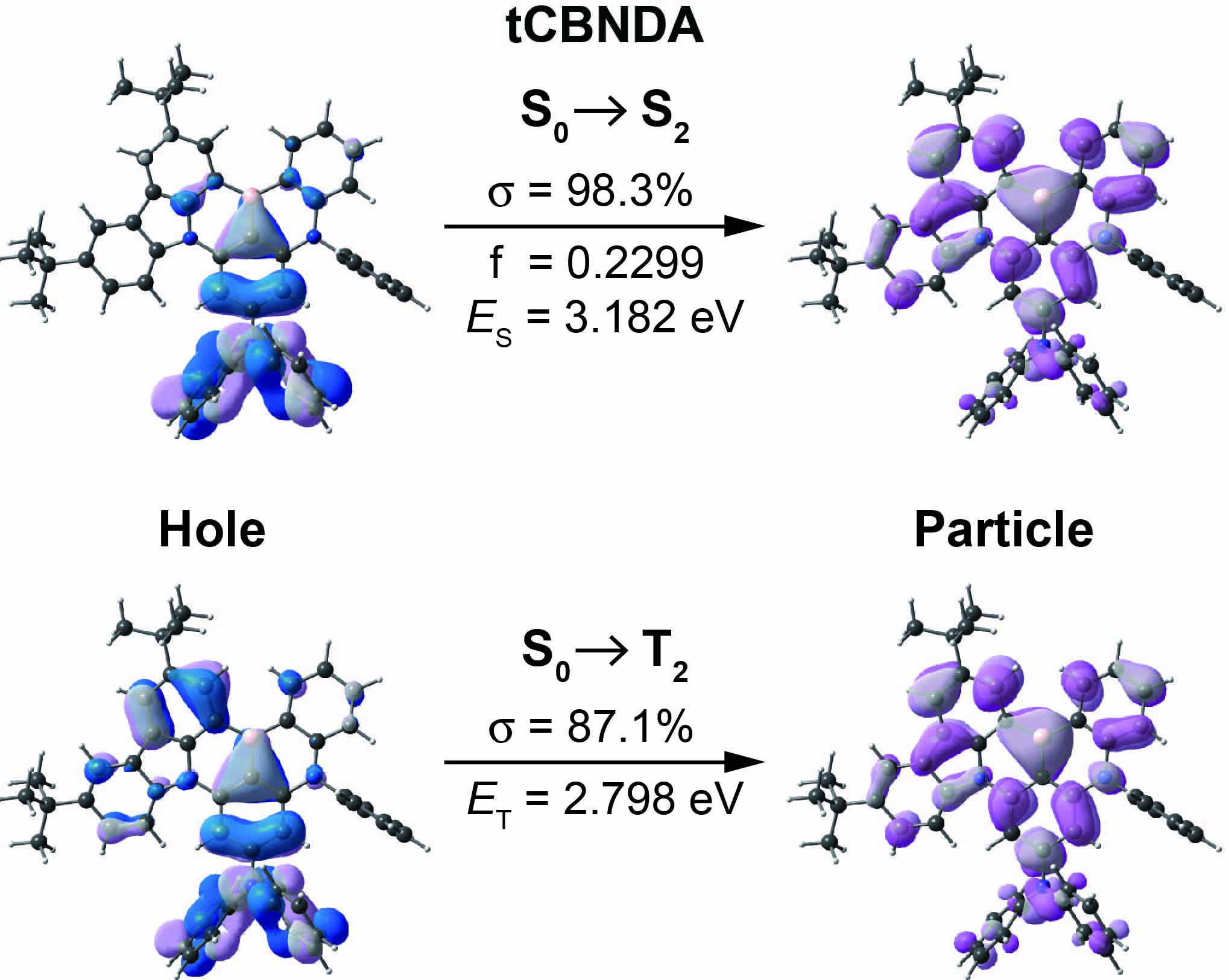


**Figure S5.** Contours of “hole” and “particle” of S_0_→S_2_ and S_0_→T_2_ excitations for **tCBNDA** simulated with the TD-DFT method. *E*_S_, *E*_T_, *f* and σ refer to the energy levels of the S_2_ and the T_2_ states, singlet oscillator strength and contribution weight.


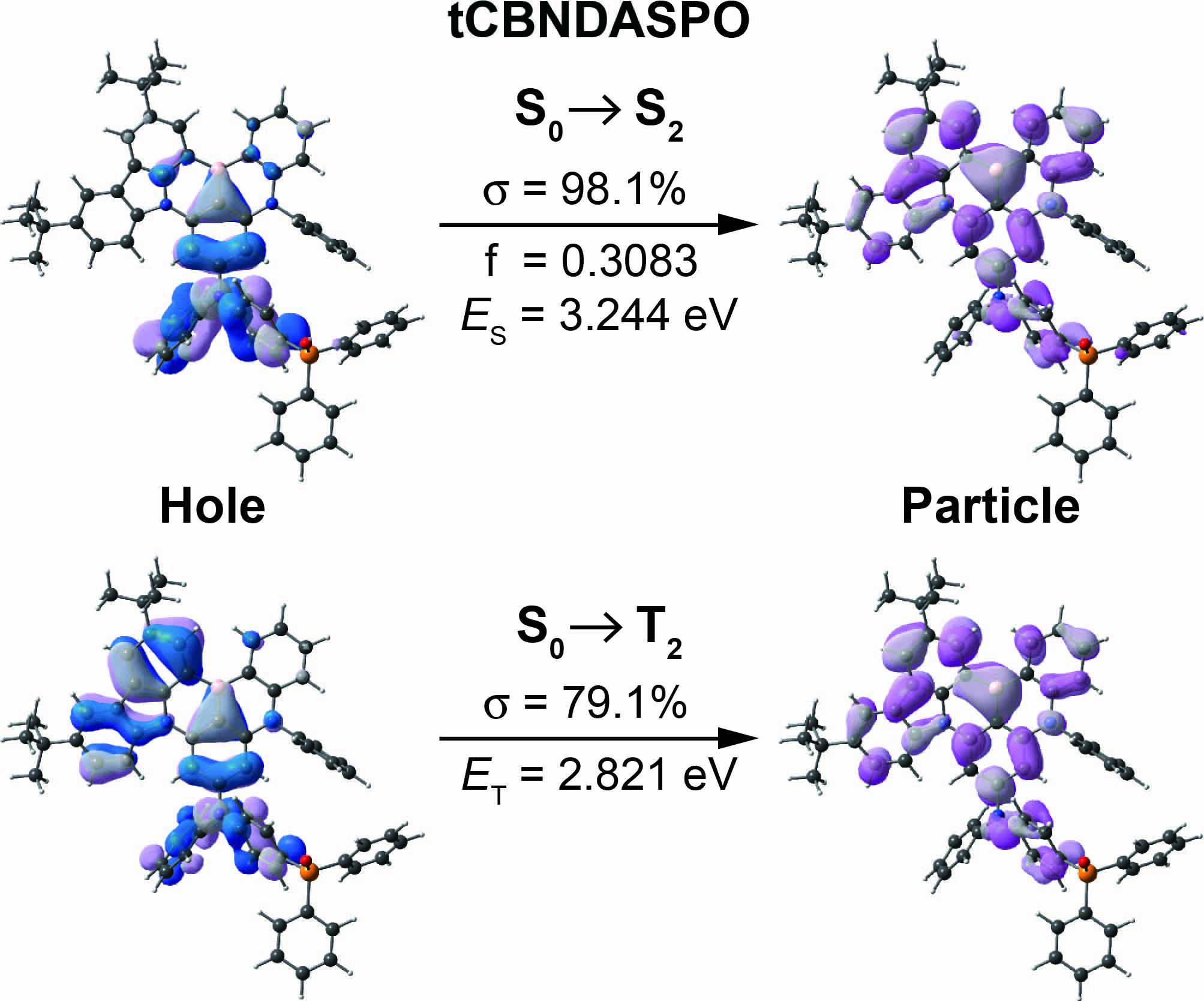


**Figure S6.** Contours of “hole” and “particle” of S_0_→S_2_ and S_0_→T_2_ excitations for **tCBNDASPO** simulated with the TD-DFT method. *E*_S_, *E*_T_, *f* and σ refer to the energy levels of the S_2_ and the T_2_ states, singlet oscillator strength and contribution weight.


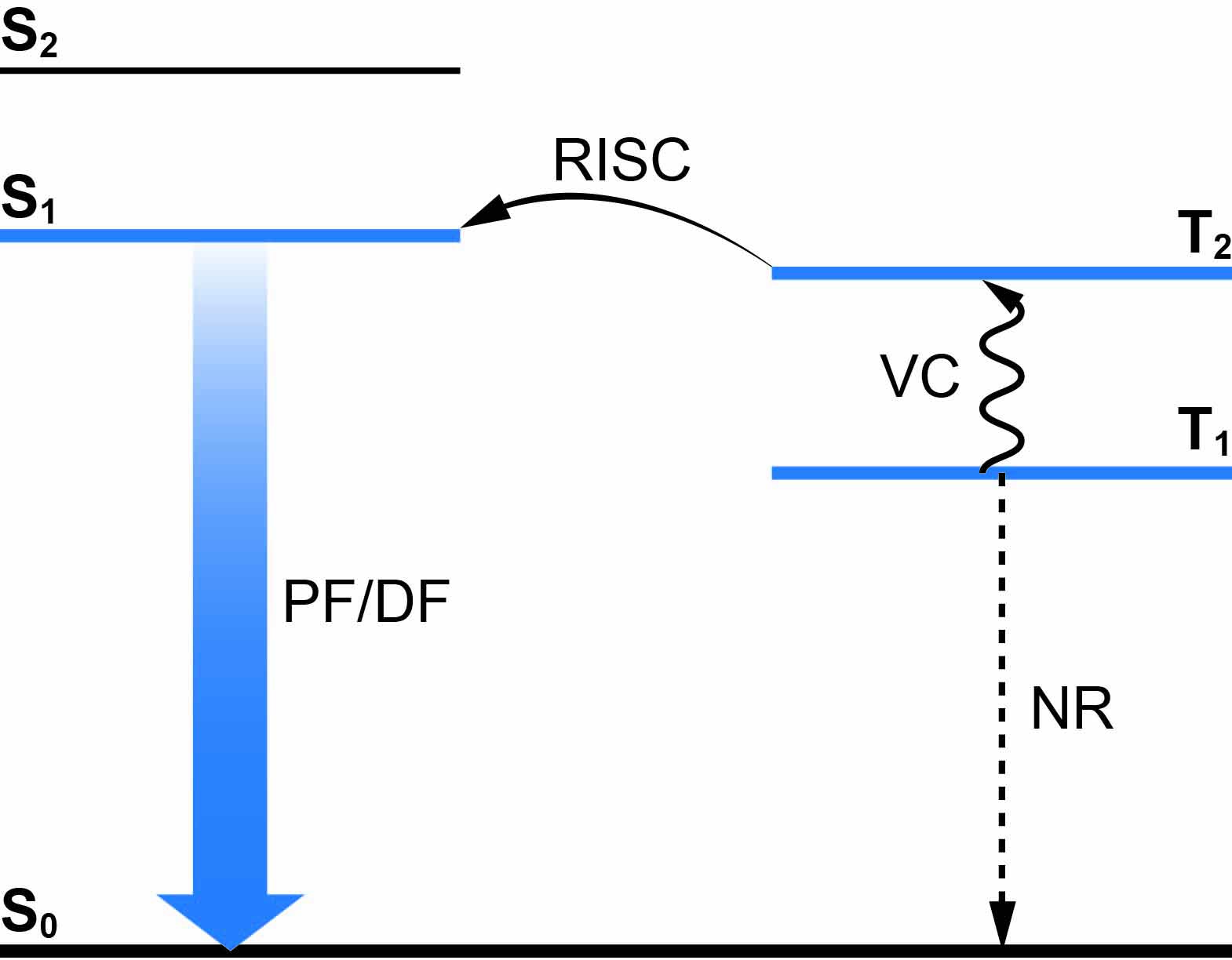


**Figure S7.** Illustation of DF processes for **tCBNDA** and **tCBNDASPO**. VC and NR refer to vibrational coupling and nonradiation.

### III. Electrochemical properties


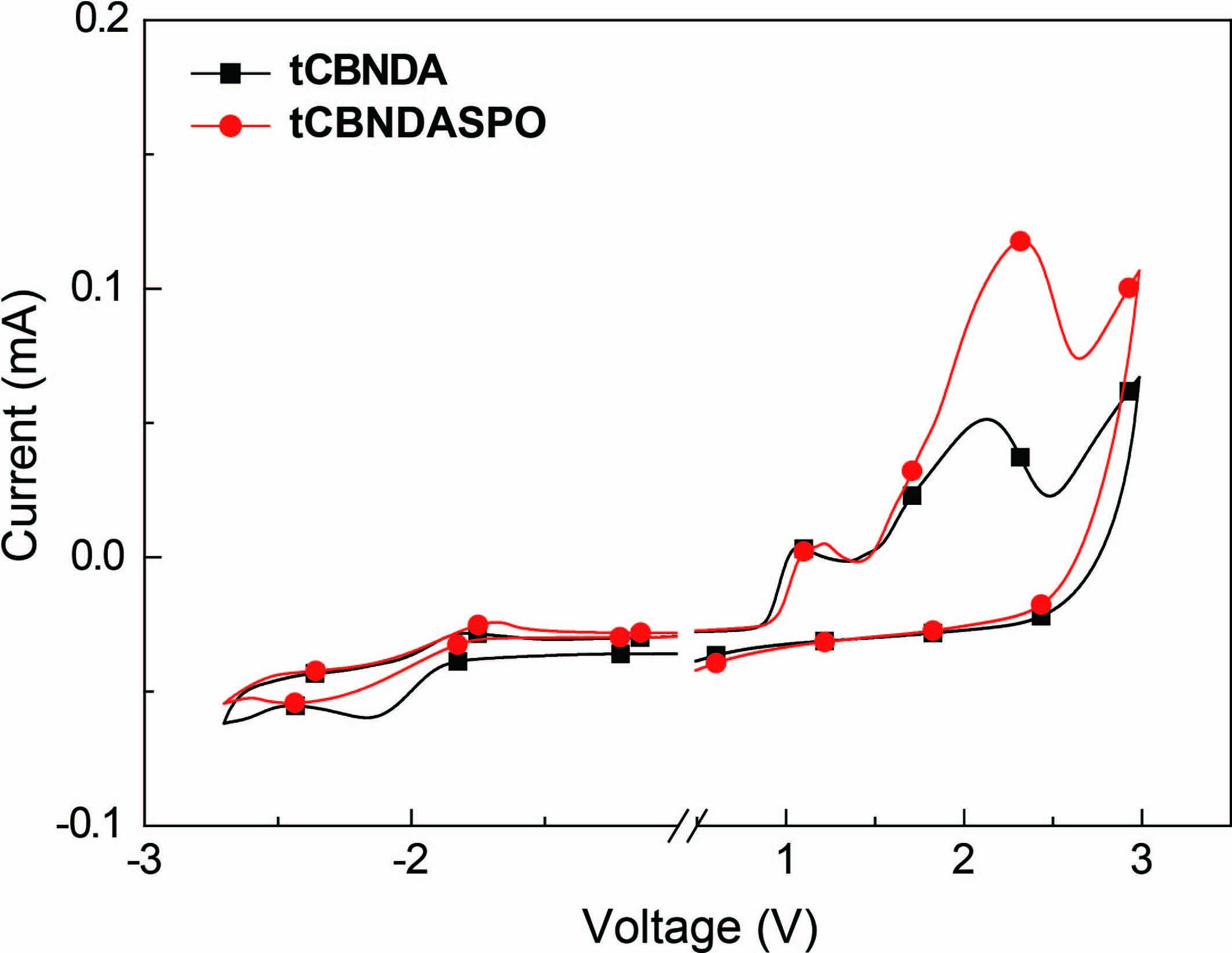


**Figure S8.** Cyclic voltammogram of **tCBNDA** and **tCBNDASPO** measured in DCM for oxidation and THF for reduction at room temperature with the scanning rate of 100 mV s^-1^ and tetra-*n*-butylammonium hexafulorophosphate as supporting electrolyte (0.1 mol L^-1^).

### IV. Photophysical properties

**
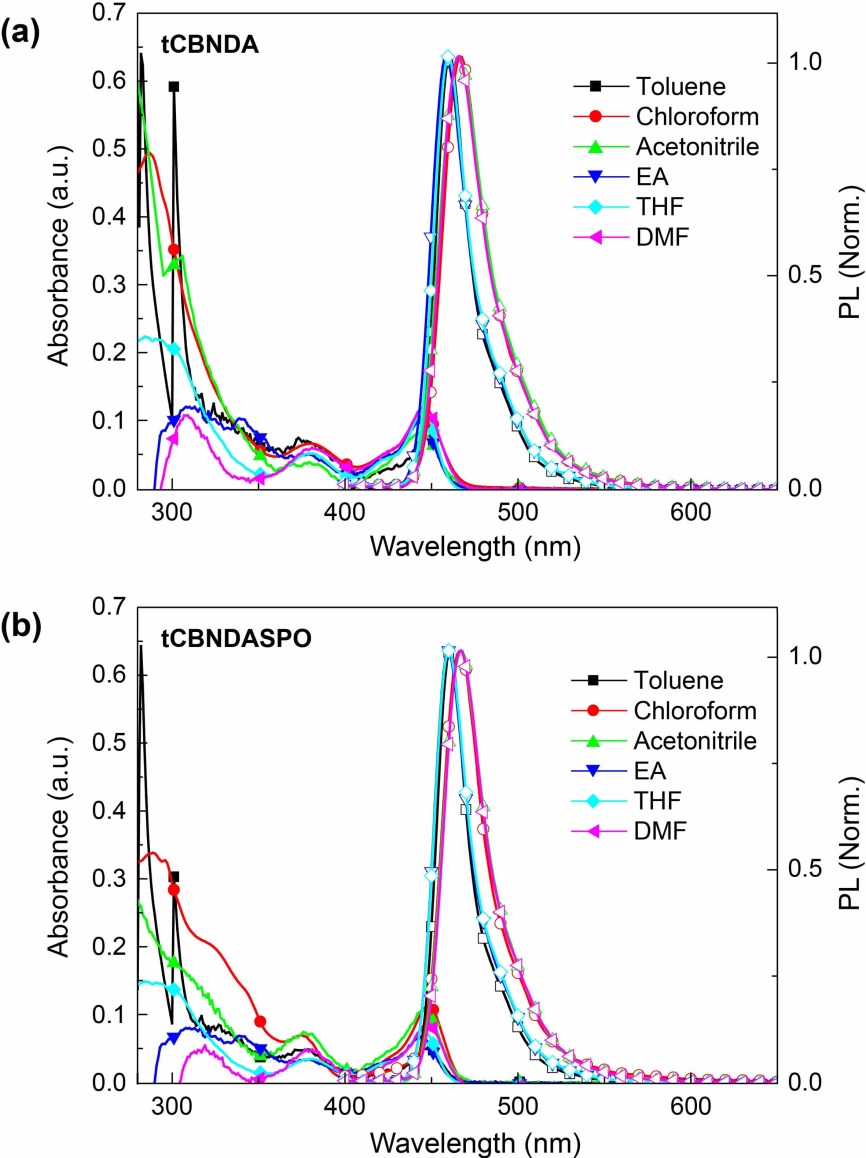
**

**Figure S9.** Electronic absorption and photoluminescence (PL) spectra of (a) **tCBNDA** and (b) **tCBNDASPO** in different solvents with diverse polarities.

**
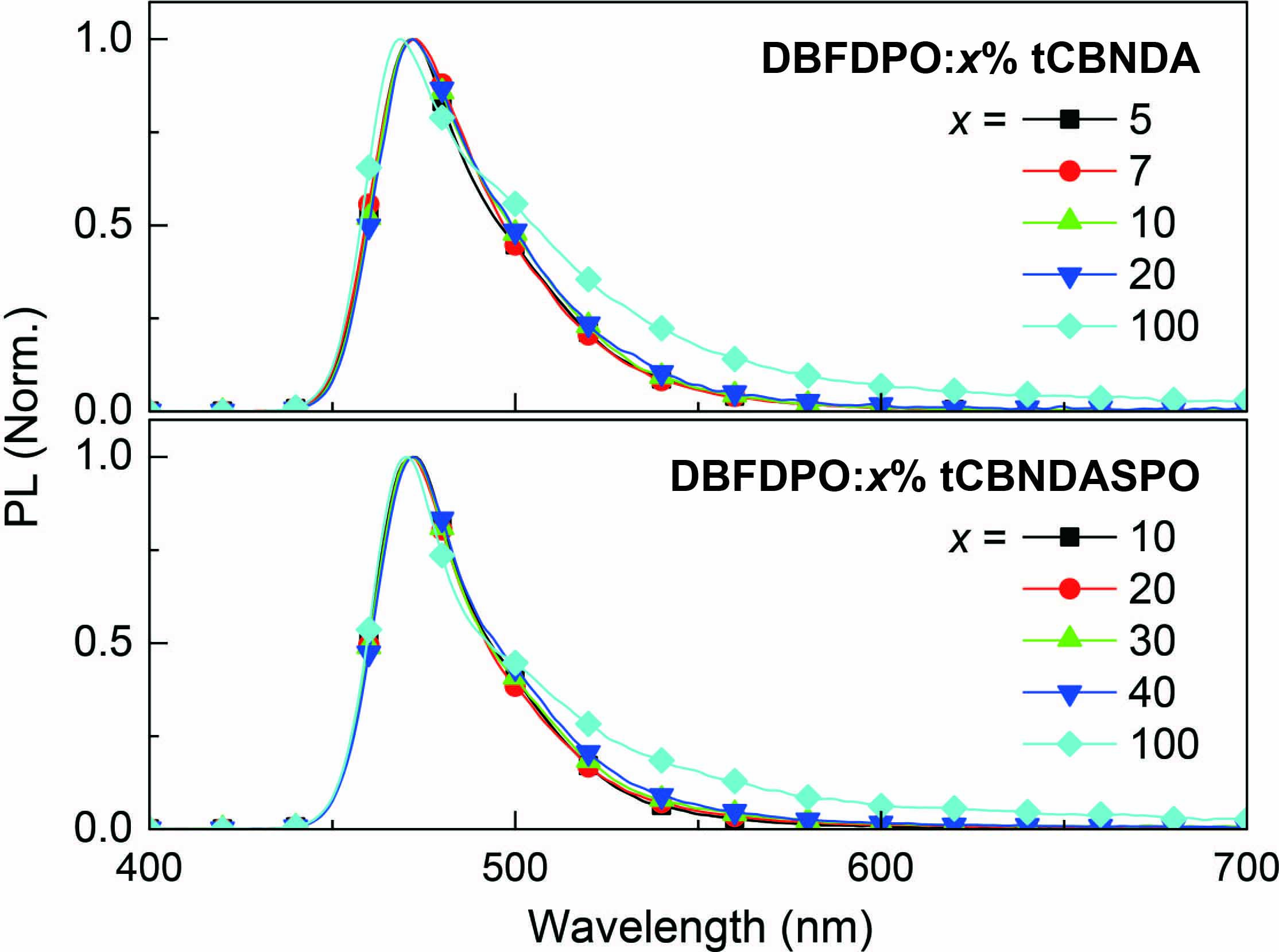
**

**Figure S10.** Variation of PL spectra for DBFDPO:*x*% **tCBNDA** and DBFDPO:*x*% **tCBNDASPO** films.

**
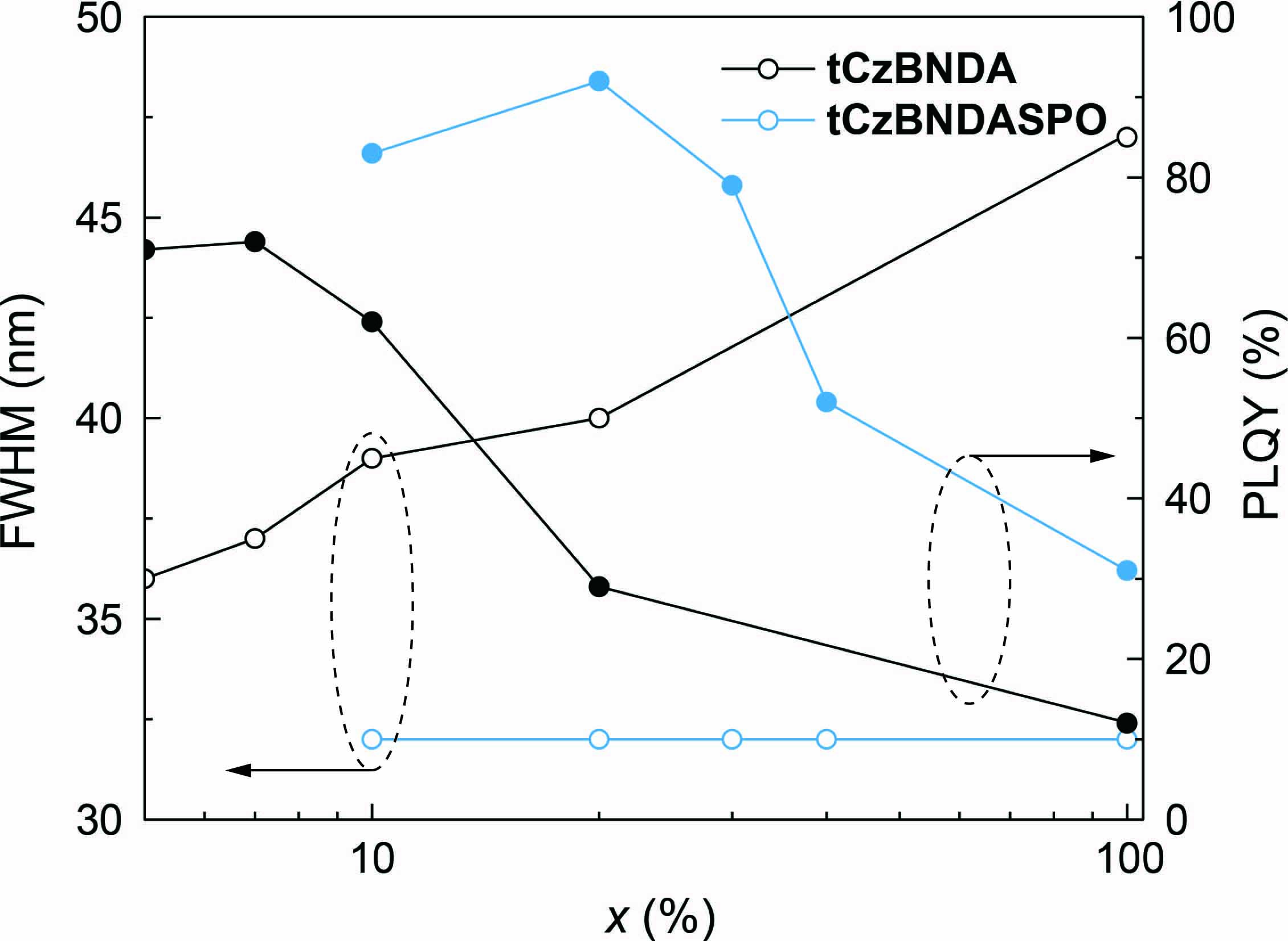
**

**Figure S11.** Dependence of FWHM and PLQY values for DBFDPO:*x*% **tCBNDA** and DBFDPO:*x*% **tCBNDASPO** films on doping concentration *x*%.

**
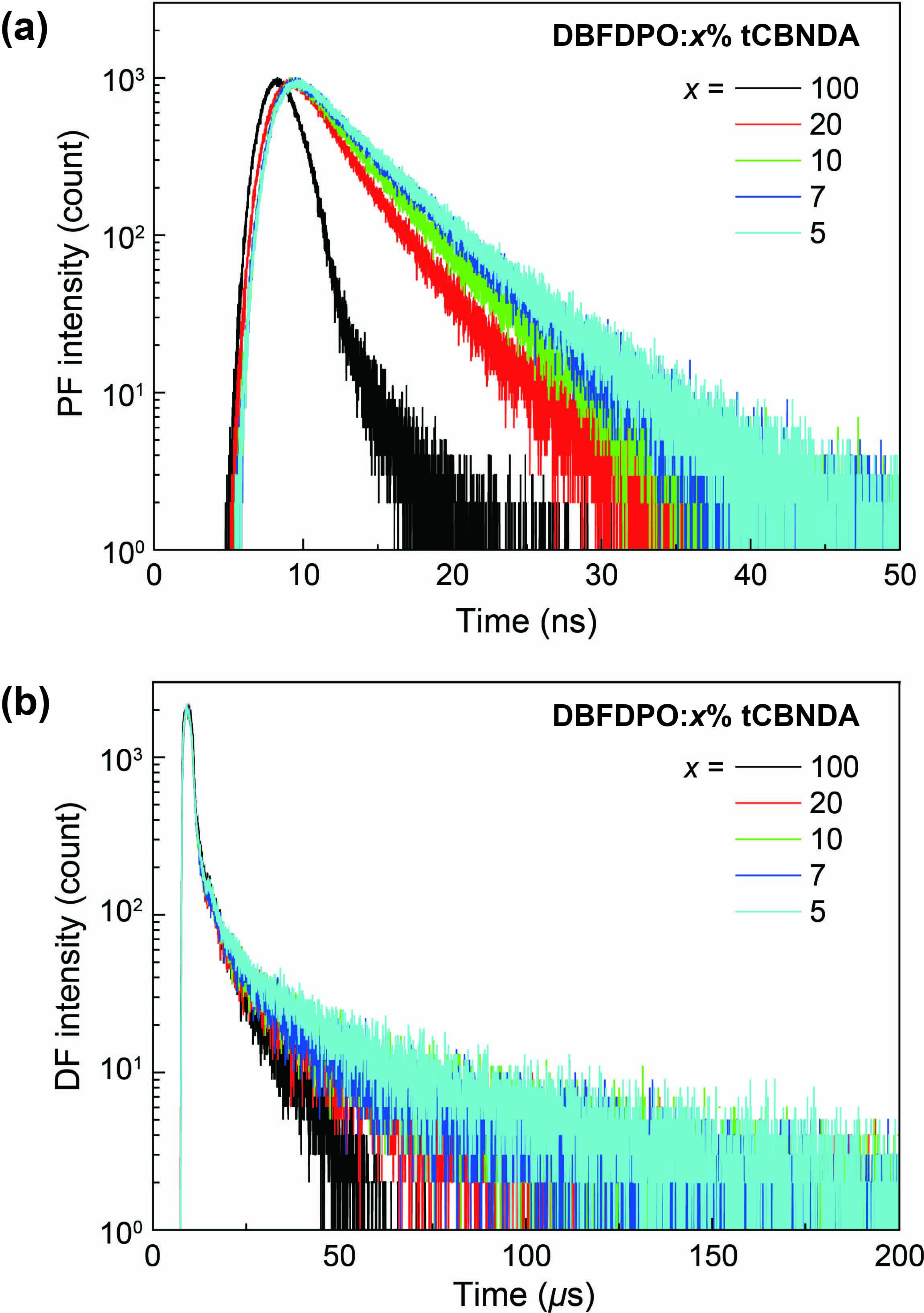
**

**Figure S12.** Concentration dependence of PF (a) and DF (b) decay curves for DBFDPO:*x*% **tCBNDA** films.

**
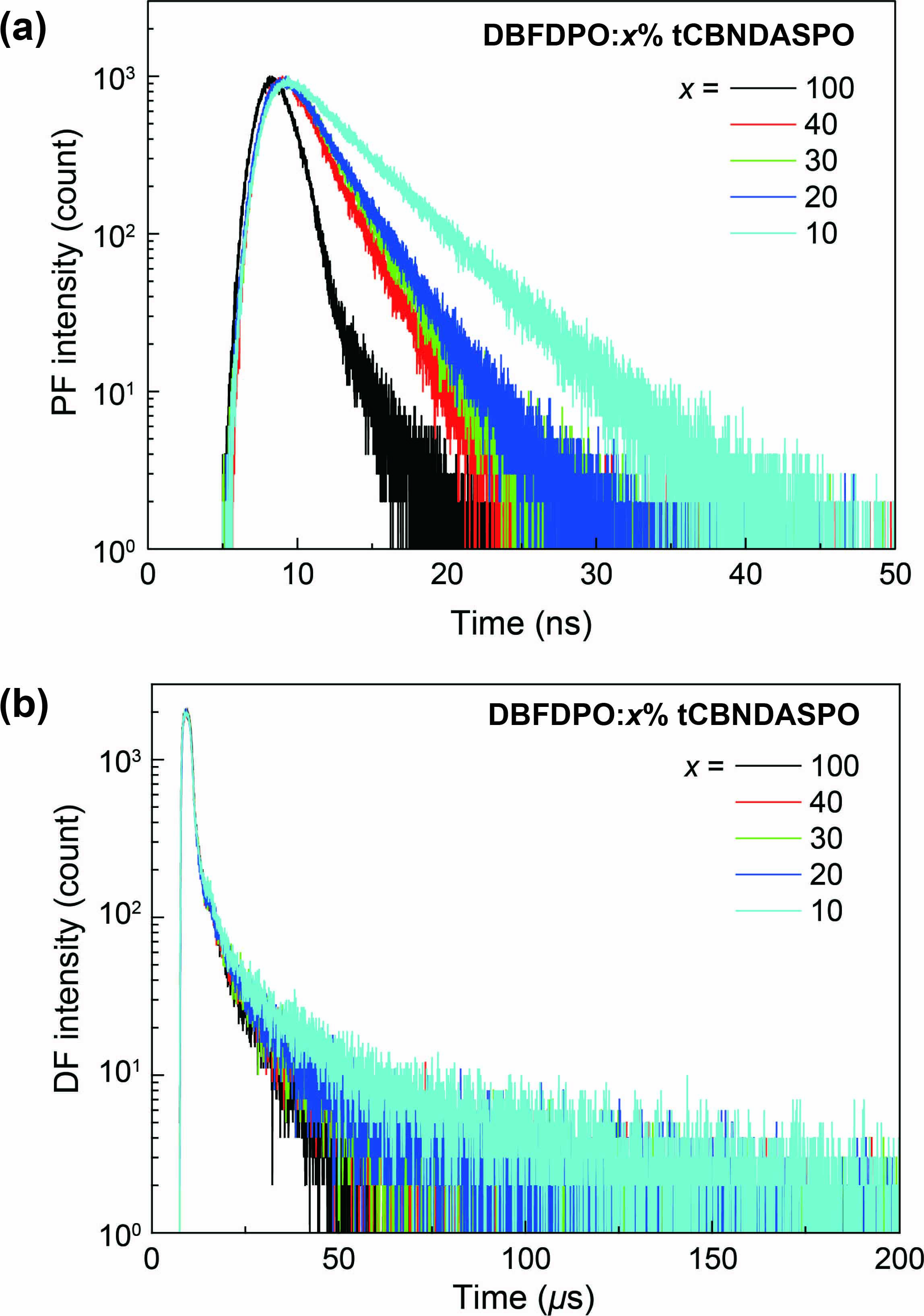
**

**Figure S13.** Concentration dependence of PF (a) and DF (b) decay curves for DBFDPO:*x*% **tCBNDASPO** films.

**
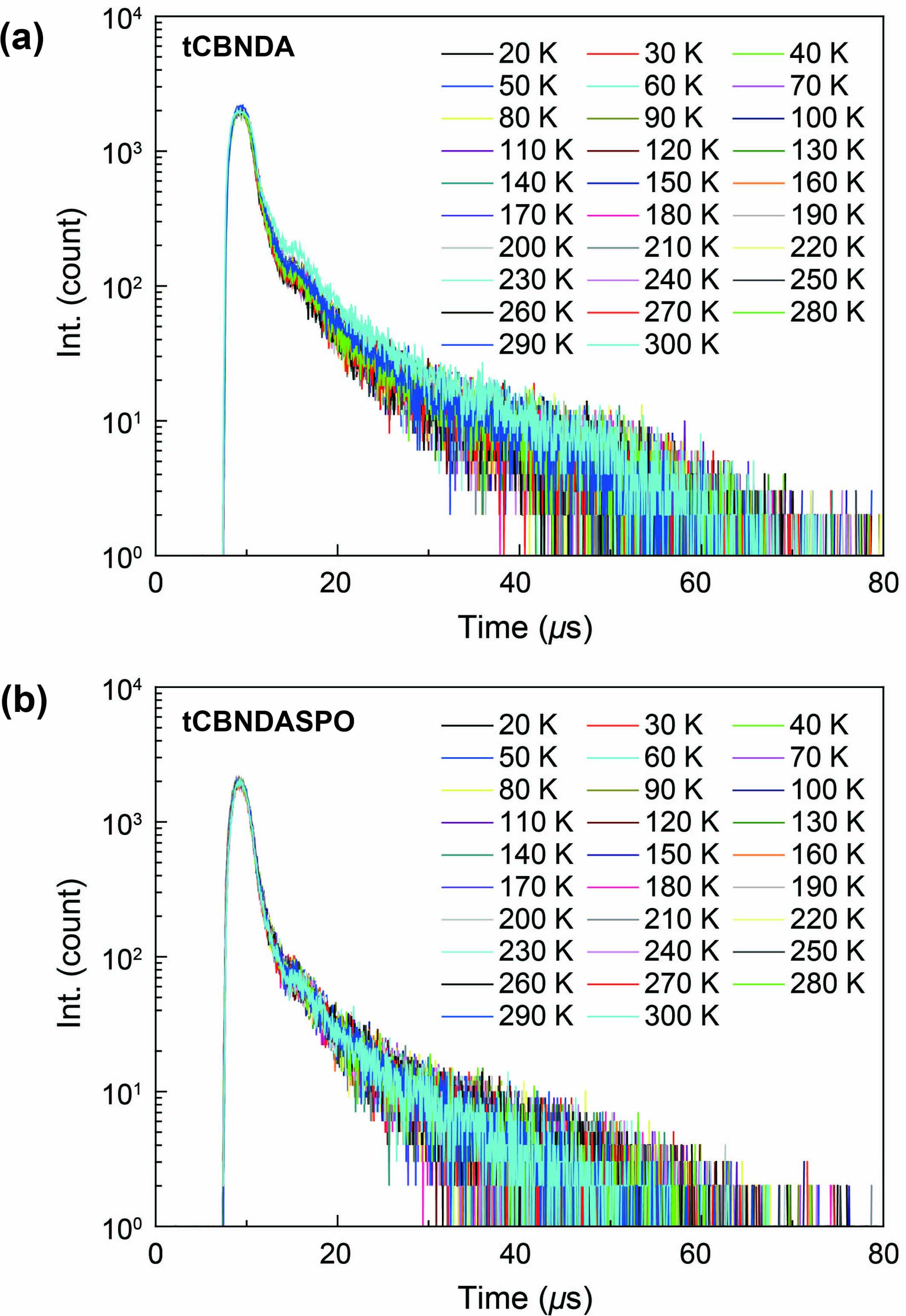
**

**Figure S14.** Temperature dependence of DF decays for neat **tCBNDA** (a) and **tCBNDASPO** (b) films.

**
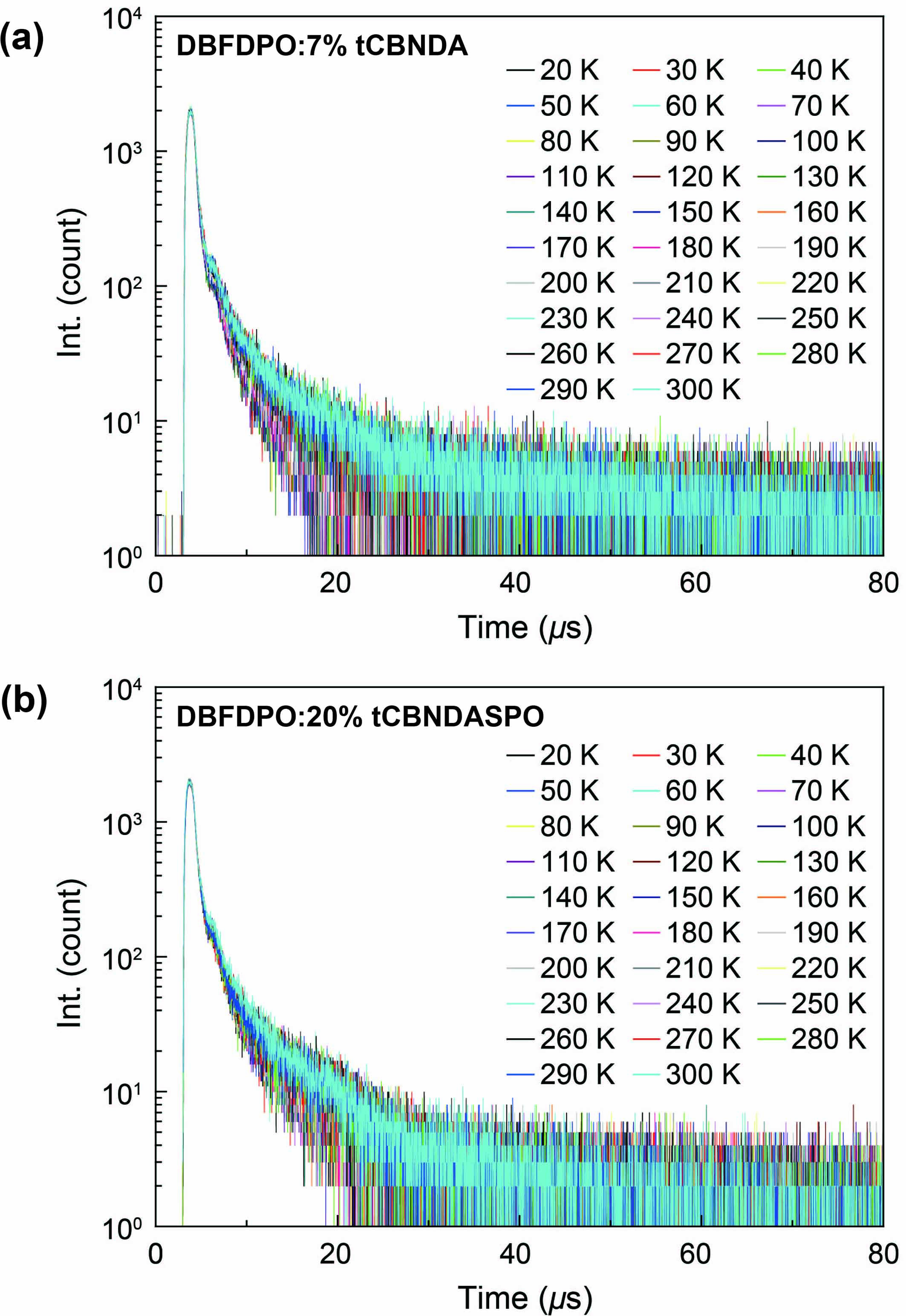
**

**Figure S15.** Temperature dependence of DF decays for DBFDPO:7% **tCBNDA** (a) and DBFDPO:20% **tCBNDASPO** (b) films.

**
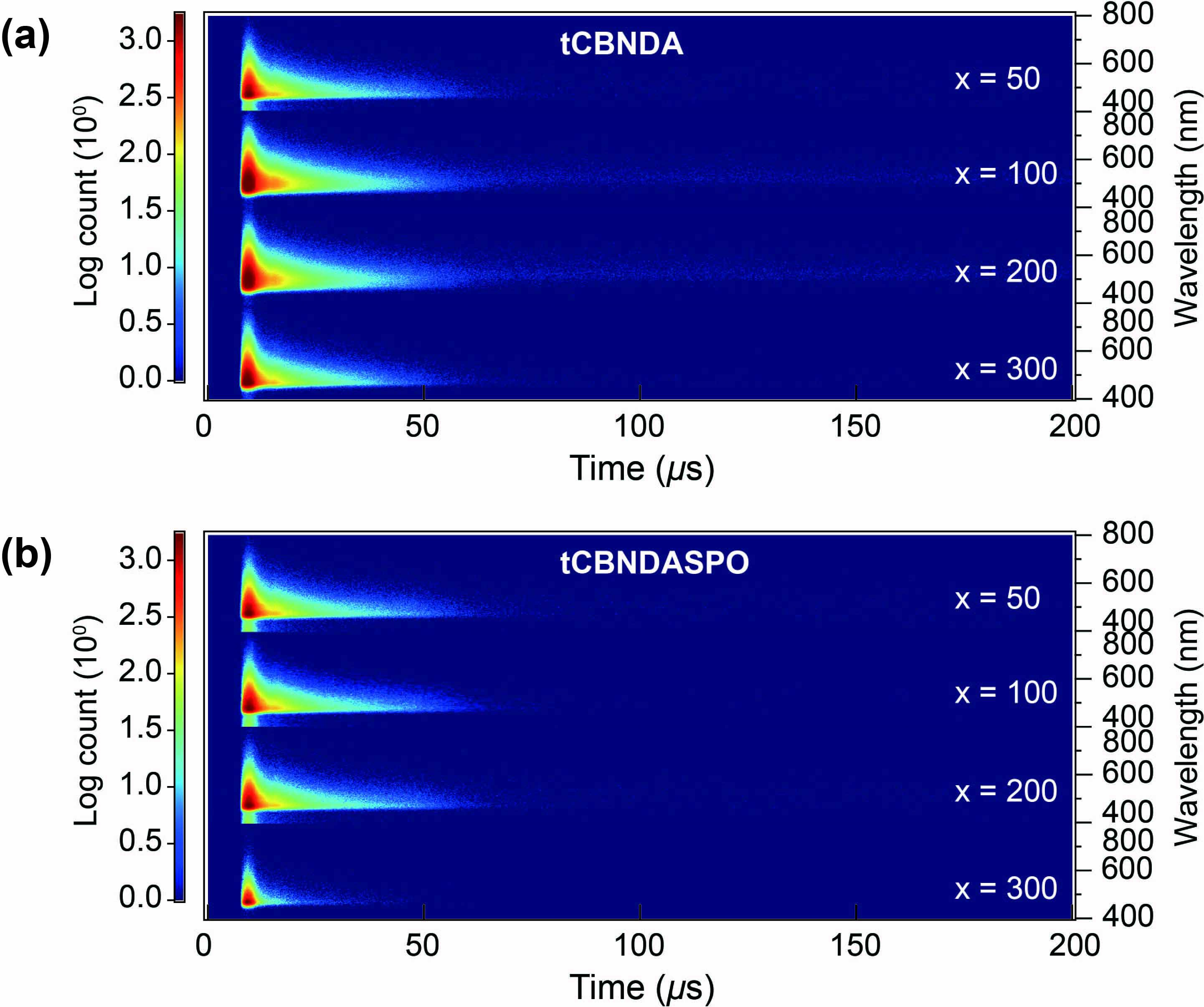
**

**Figure S16.** Temperature dependence of time-resolved emission spectra (TRES) for neat **tCBNDA** (a) and **tCBNDASPO** (b) films.

**
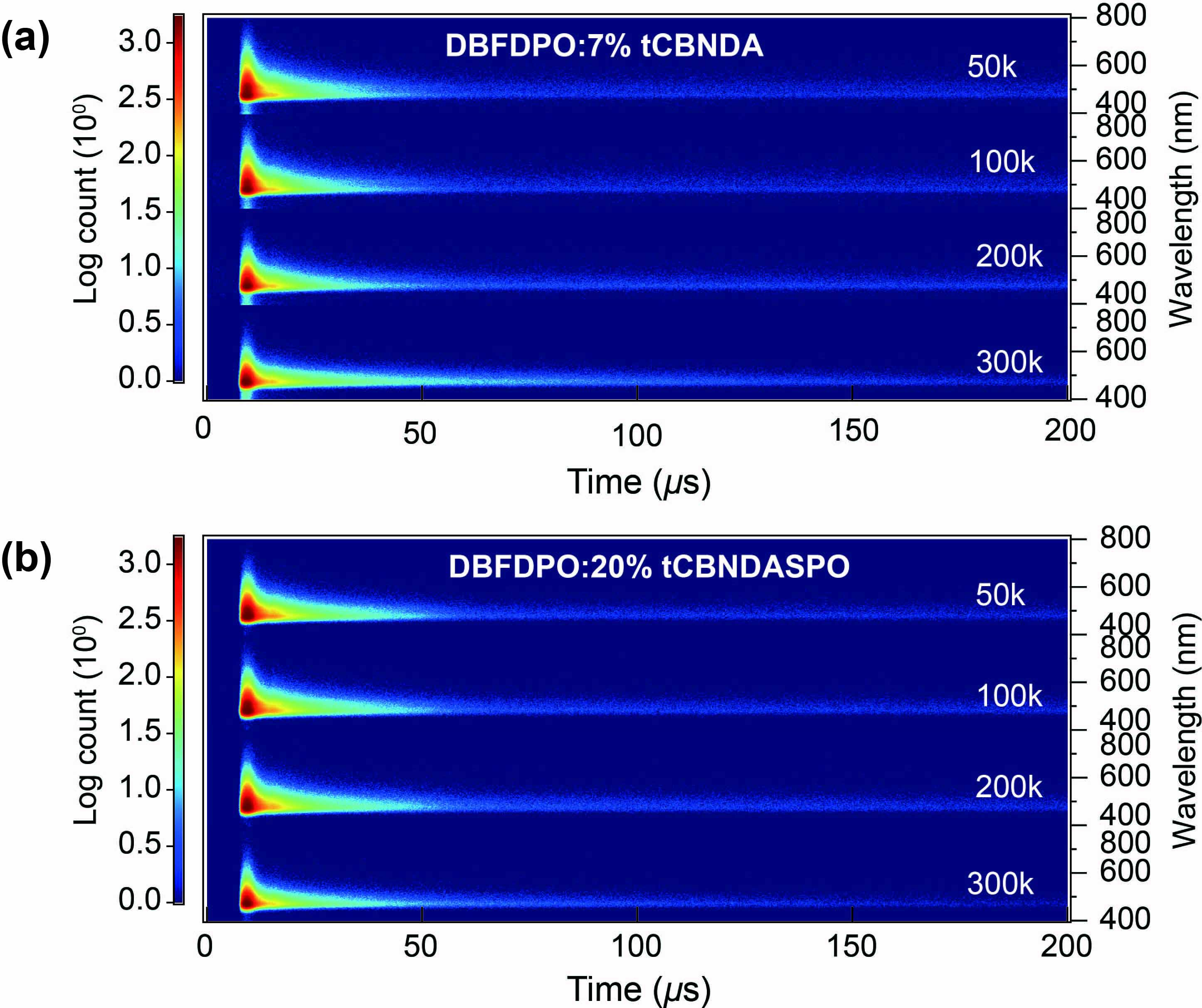
**

**Figure S17.** Temperature dependence of time-resolved emission spectra (TRES) for DBFDPO:7% **tCBNDA** (a) and DBFDPO:20% **tCBNDASPO** (b) films.

**
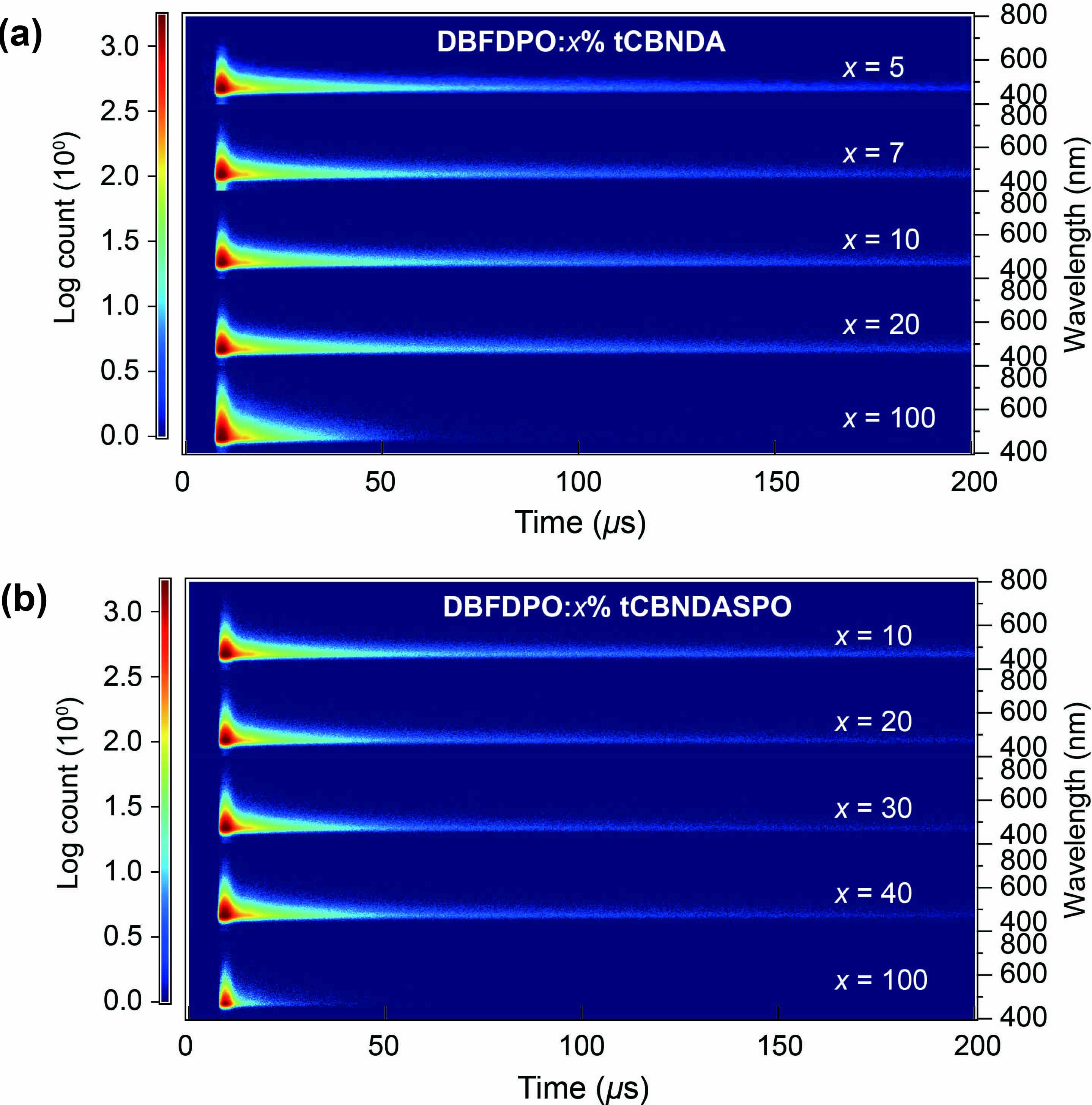
**

**Figure S18.** Concentration dependence of time-resolved emission spectra (TRES) for DBFDPO:*x*% **tCBNDA** (a) and DBFDPO:*x*% **tCBNDASPO** (b) films.

### V. Morphological properties


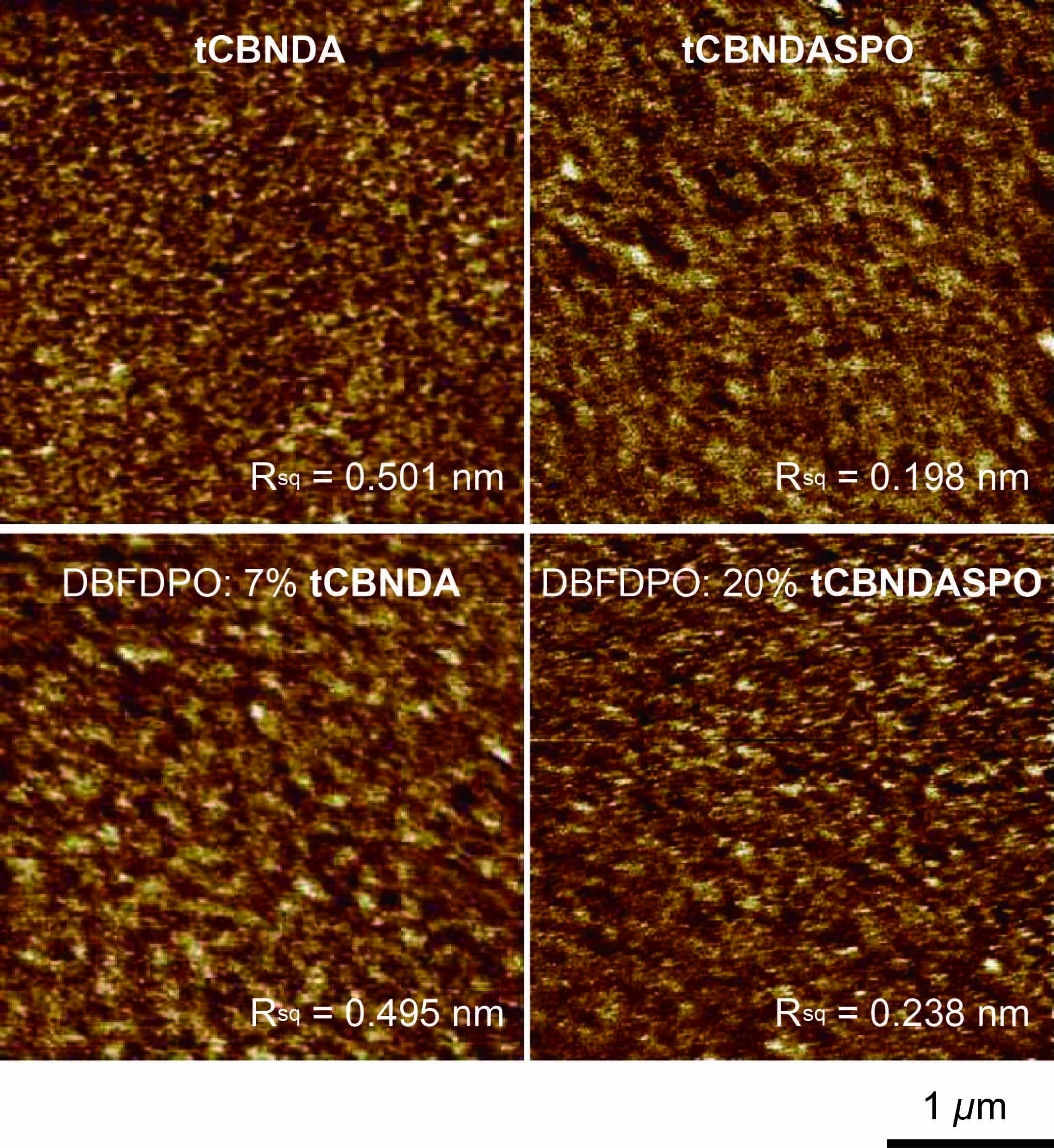


**Figure S19.** Atom force microscopy (AFM) images of neat films and DBFDPO:7% **tCBNDA** and DBFDPO:20% **tCBNDASPO** films.

### VI. OLED performances


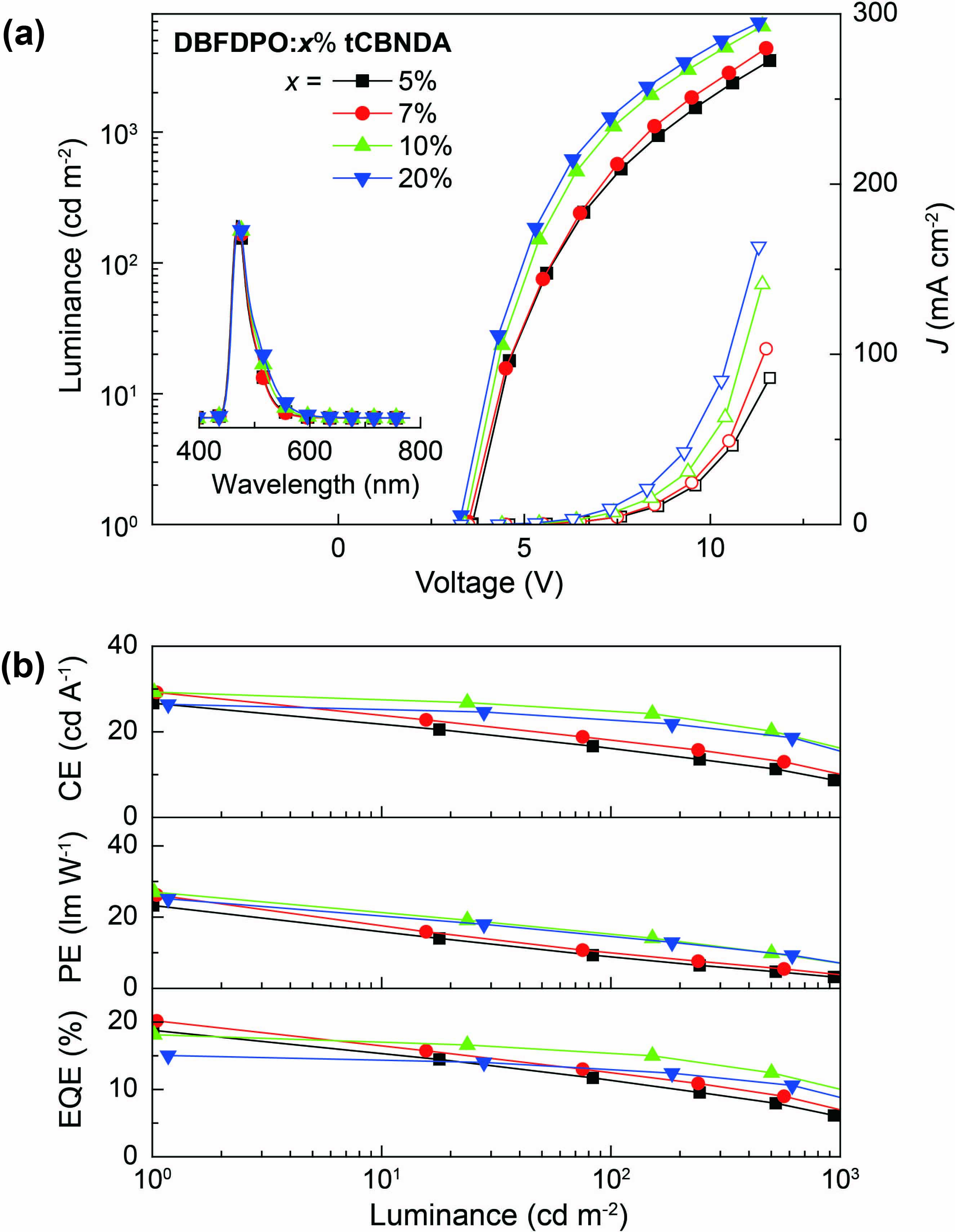


**Figure S20.** (a) EL spectra (inset) and luminance-current density (*J*)-voltage curves of the devices with diffferent **tCBNDA** doping concentrations of 5%, 7%, 10% and 20%; (b) efficiencies *vs.* luminance relationships of the devices.


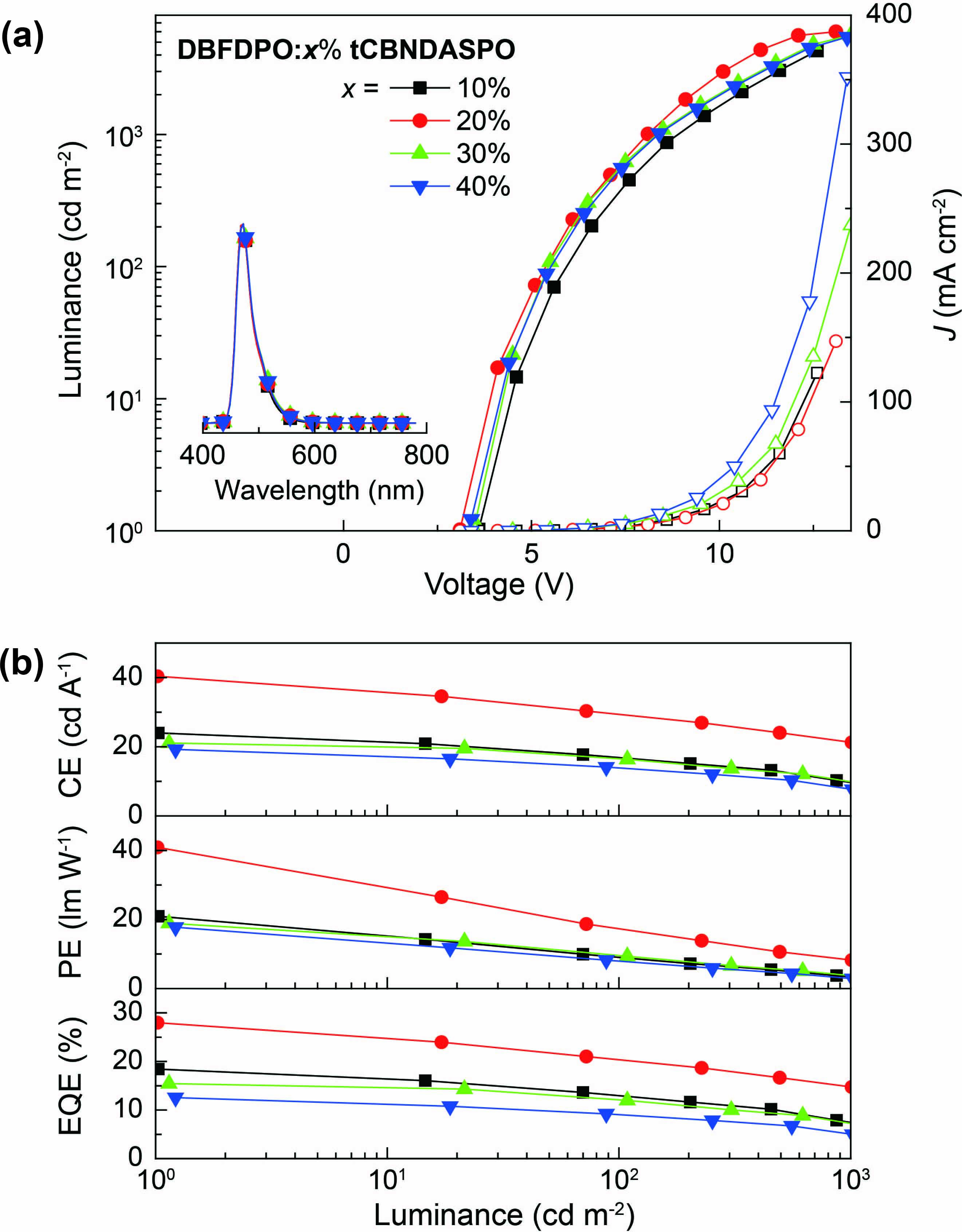


**Figure S21.** (a) EL spectra (inset) and luminance-current density (*J*)-voltage curves of the devices with diffferent **tCBNDASPO** doping concentrations of 10%, 20%, 30% and 40%; (b) efficiencies *vs.* luminance relationships of the devices.

### Table S1. Basic physical properties of tCBNDA and tCBNDASPO.

|  | *λ*_Abs._ (nm) | *λ*_PL_  (nm) | FWHM  (nm) | S_1_  (eV) | T_1_  (eV) | Δ*E*_ST_^[f]^  (eV) | *f*_S_ | *T*_m_ */ T*_d_  (^o^C) | HOMO  (eV) | LUMO  (eV) |
| --- | --- | --- | --- | --- | --- | --- | --- | --- | --- | --- |
| **tCBNDA** | 230, 288, 380, 446^[a]^  225, 280, 450^[b]^ | 467^[a]^  470^[b]^ | 28^[a]^  47^[b]^ | 3.00^[c]^  2.62^[d]^ | 2.57^[c]^  2.57^[e]^ | 0.43^[c]^  0.05 | 0.2908^[c]^  0.3417^[g]^ | 272, 381^[h]^ | -4.89^[c]^  -5.67^[i]^ | -1.39^[c]^  -2.95^[i]^ |
| **tCBNDASPO** | 230, 285, 325, 380, 448^[a]^  225, 285, 450 ^[b]^ | 467^[a]^  470^[b]^ | 28^[a]^  32^[b]^ | 2.99^[c]^  2.62^[d]^ | 2.55^[c]^  2.58^[e]^ | 0.43^[c]^  0.04 | 0.2929^[c]^  0.3738^[g]^ | 285, 447^[h]^ | -4.94^[c]^  -5.74^[i]^ | -1.46^[c]^  -2.99^[i]^ |

[a] In DCM solution (10-6 mol L-1); [b] in film; [c] Gaussian simulation results; [d] estimated according to absorption edge; [e] estimated according to 0-0 transition in phosphorescence spectrum; [f] singlet-triplet splitting; [g] estimated according to absorption spectrum; [h] temperature at weight loss of 5%; [i] calculated according to cyclic voltammetric results.

### Table S2. TADF characteristics of DBFDPO:*x*% tCBNDA and DBFDPO:*x*% tCBNDASPO films.

| MR emitter | *x*% | *λ*_Em_  (nm) | FWHM^[a]^  (nm) | *ϕ*_PL_^[b]^  (%) | *ϕ*_PF_^[c]^  (%) | *ϕ*_DF_^[d]^  (%) | *τ*_PF_^[e]^  (ns) | *τ*_DF_^[f]^  (*µ*s) | *k*_PF_^[g]^  (10^7^ s^-1^) | *k*_DF_^[h]^  (10^4^ s^-1^) | *k*_RISC_^[i]^  (10^4^ s^-1^) | $k_{r}^{S}$^[j]^  (10^6^ s^-1^) | $k_{nr}^{T}$^[k]^  (10^4^ s^-1^) | *Φ*_ISC_^[l]^  (%) | *Φ*_RISC_^[m]^  (%) |
| --- | --- | --- | --- | --- | --- | --- | --- | --- | --- | --- | --- | --- | --- | --- | --- |
| **tCBNDA** | 5 | 472 | 36 | 71 | 20 | 51 | 4.9 | 27.0 | 4.07 | 1.89 | 6.76 | 8.10 | 0.55 | 72 | 92 |
|  | 7 | 472 | 37 | 72 | 19 | 53 | 4.7 | 22.8 | 4.14 | 2.31 | 8.55 | 8.04 | 0.65 | 73 | 93 |
|  | 10 | 472 | 39 | 62 | 16 | 46 | 4.1 | 22.8 | 3.92 | 2.02 | 7.79 | 6.32 | 0.77 | 74 | 91 |
|  | 20 | 472 | 40 | 29 | 20 | 9 | 3.3 | 19.3 | 6.08 | 0.45 | 0.64 | 12.35 | 0.32 | 30 | 67 |
|  | 100 | 470 | 47 | 12 | 10 | 2 | 1.3 | 6.6 | 7.47 | 0.35 | 0.43 | 7.26 | 0.30 | 19 | 58 |
| **tCBNDASPO** | 10 | 472 | 32 | 83 | 54 | 29 | 4.7 | 19.5 | 11.59 | 1.49 | 2.30 | 62.53 | 0.25 | 35 | 90 |
|  | 20 | 472 | 32 | 92 | 63 | 29 | 2.9 | 9.1 | 22.07 | 3.14 | 4.55 | 140.11 | 0.25 | 31 | 95 |
|  | 30 | 472 | 32 | 79 | 56 | 23 | 2.6 | 8.4 | 21.30 | 2.71 | 3.82 | 119.45 | 0.57 | 29 | 87 |
|  | 40 | 472 | 32 | 52 | 37 | 15 | 2.3 | 8.3 | 15.77 | 1.82 | 2.56 | 58.24 | 0.87 | 29 | 75 |
|  | 100 | 470 | 32 | 31 | 24 | 7 | 1.2 | 7.1 | 19.24 | 1.05 | 1.38 | 45.33 | 0.72 | 24 | 66 |

[a] Full width at half maximum; [b] Absolute PL quantum yield measured with integrating sphere; quantum efficiencies of prompt fluorescence (PF) [c] and delayed fluorescence (DF) [d]; lifetimes of PF [e] and DF[f]; rate constants of PF [g], DF[h], reverse intersystem crossing (RISC) [i], singlet radiation [j] and triplet nonradiation [k]; quantum efficiencies of intersystem crossing (ISC) [l] and RISC [m].

### Table S3. EL performance of DBFDPO:*x*% tCBNDA and DBFDPO:*x*% tCBNDASPO.

| MR emitter | *x* (%) | *V*^[a]^  (V) | *L*_max_^[b]^  (cd m^-2^) | *η*^[c]^ | | | *λ*_EL_ / FWHM (nm)  CIE (x, y)^[d]^ |
| --- | --- | --- | --- | --- | --- | --- | --- |
|  |  |  |  | *η*_CE_ (cd A^-1^) | *η*_PE_ (lm W^-1^) | *η*_EQE_ (%) |  |
| **tCBNDA** | 5 | 3.6, 5.8, 8.7 | 3520 | 26.66, 15.99, 8.14 | 23.25, 8.89, 2.83 | 18.77, 11.22, 5.71 | 468 / 36  (0.12, 0.17) |
|  | 7 | 3.5, 5.7, 8.3 | 4370 | 29.19, 18.01, 10.16 | 26.19, 10.71,9.35 | 20.17, 12.44, 7.13 | 472 / 36  (0.12, 0.17) |
|  | 10 | 3.4, 5.1, 7.2 | 6446 | 29.26, 24.95 ,15.19 | 27.03, 15.44, 6.98 | 18.11,15.53, 10.08 | 472 / 36  (0.12, 0.20) |
|  | 20 | 3.3, 4.9, 6.9 | 6857 | 26.41,22.73 , 15.29 | 25.13, 14.42, 6.98 | 15.03, 12.84, 8.8 | 472 / 40  (0.13, 0.23) |
| **tCBNDASPO** | 10 | 3.6, 5.9, 8.9 | 4309 | 23.99, 17.10, 9.73 | 20.92,9.06, 2.64 | 18.42,12.99, 7.51 | 472 / 32  (0.12, 0.17) |
|  | 20 | 3.1, 5.3, 8.1 | 5997 | 40.38, 29.81, 21.77 | 40.90,17.34,8.63 | 27.98, 20.64, 15.27 | 472 / 32  (0.12, 0.17) |
|  | 30 | 3.5, 5.4, 8.3 | 5657 | 21.06, 16.60, 9.88 | 18.92, 9.64, 3.78 | 15.44, 11.74, 7.15 | 472 / 32  (0.12, 0.18) |
|  | 40 | 3.6, 5.8, 8.7 | 3520 | 26.66, 15.99, 8.14 | 23.25, 8.89, 2.83 | 18.77, 11.22, 5.71 | 472 / 32  (0.12, 0.18) |

[a] At 1, 100 and 1000 cd m^-2^; [b] the maximum luminance; [c] EL efficiencies at the maximum and 100 and 1000 cd m^-2^; [d] peak wavelengths and CIE coordinates of EL emissions at 1000 cd m^-2^.

### Table S4. EL performance of representative functionalized MR-TADF emitters.

| Device structure | Emitter | *V_on_*^[a]^ | *L*_max_^[b]^  (cd m^-2^) | *η*^[c]^ | | | *λ*_EL_ / FWHM (nm)  CIE (x, y) | Ref. |
| --- | --- | --- | --- | --- | --- | --- | --- | --- |
|  |  |  |  | *η*_CE_ (cd A^-1^) | *η*_PE_ (lm W^-1^) | *η*_EQE_ (%) |  |  |
| ITO\|MoO3(2.5 nm) \|TAPC (30nm) \|2,6-DCzppy :4 wt%TBN-TPA(10 nm) \|TmPyPB (30nm) \|LiF (1 nm) \|Al (100 nm) |  | 3.5  ≈4.1  ≈4.8 | 16593 | 40.2  34.4  17.4 | 30.0  23.5  8.8 | 32.1  27.4  13.9 | 474/27 nm  (0.12,0.19) | (*6*) |
| ITO\|MoO3 (10 nm) \|TAPC (60 nm) \| mCP (5 nm) \| mCP: 3 wt%PAB (20 nm) \| DPEPO (15 nm) \| TmPyPB (30 nm) \| /LiF (1 nm )\|Al(100 nm) |  | 4.7  ≈7.8  - | 782 | 10.4  ≈6.0  - | 7.1  ≈2.5  - | 14.7  ≈9.0  - | 456/31 nm  (0.145, 0.076) | (*7*) |
| ITO\|MoO3 (10 nm) \|TAPC (60 nm) \| mCP (5 nm) \| mCP: 3 wt% 2tPAB (20 nm) \| DPEPO (15 nm) \| TmPyPB (30 nm) \| /LiF (1 nm )\|Al(100 nm) |  | 4.5  ≈7.0  ≈12.5 | 1241 | 11.8  ≈8.0  ≈2.8 | 8.0  ≈3.0  ≈0.8 | 16.8  ≈12.0  ≈3.0 | 456/27 nm  (0.145, 0.076) | (*7*) |
| ITO\|MoO3 (10 nm) \|TAPC (60 nm) \| mCP (5 nm) \| mCP: 3 wt% 3tPAB (20 nm) \| DPEPO (15 nm) \| TmPyPB (30 nm) \| /LiF (1 nm )\|Al(100 nm) |  | 4.1  ≈6.8  ≈12.5 | 1100 | 13.4  ≈10.0  ≈3.0 | 10.2  ≈8.0  ≈0.9 | 19.3  ≈15.0  ≈3.0 | 460/26 nm  (0.141, 0.076) | (*7*) |
| ITO\| TADPC (50 nm) \| mCBP (10 nm) \| mCBP: BBCz-DB,2 wt% (20 nm) \| PPF (10 nm) \|B3PYPB (40 nm) \| LiF \| Al |  | -  -  - | - | -  -  - | -  -  - | 29.3  -  - | 469/27 nm  (0.12, 0.18) | (*8*) |
| ITO\| TADPC (50 nm) \| mCBP (10 nm) \| mCBP: BBCz-G,2 wt% (20 nm) \| PPF (10 nm) \|B3PYPB (40 nm) \| LiF \| Al |  | -  -  - | - | -  -  - | -  -  - | 31.8  -  - | 515/54 nm  (0.26, 0.68) | (*8*) |
| ITO\| TADPC (50 nm) \| mCBP (10 nm) \| mCBP: BBCz-Y,2 wt% (20 nm) \| PPF (10 nm) \|B3PYPB (40 nm) \| LiF \| Al |  | -  -  - | - | -  -  - | -  -  - | 29.3  -  - | 549/48 nm  - | (*8*) |
| ITO\| TADPC (50 nm) \| mCBP (10 nm) \| mCBP: BBCz-R,2 wt% (20 nm) \| PPF (10 nm) \|B3PYPB (40 nm) \| LiF \| Al |  | -  -  - | - | -  -  - | -  -  - | 22.0  -  - | 616/26 nm  (0.67, 0.33) | (*8*) |
| ITO\| HAT-CN (5 nm) \| TAPC (30 nm) \| mCP (10 nm) \| 1 wt% T-CzB:oCBP(20 nm) \| PPF(10nm)\|B3PyPB (40nm)\| Liq \| Al |  | -  -  - | - | 100.7  -  - | 72.4  -  - | 29.2  24.7  9.4 | 515/30 nm  (0.16 0.71) | (*9*) |
| ITO\| HAT-CN (5 nm) \| TAPC (30 nm) \| mCP (10 nm) \| 1 wt% DACz-B:oCBP(20 nm) \| PPF(10nm)\|B3PyPB (40nm)\| Liq \| Al |  | -  -  - | -, | 81.6  -  - | 58.2  -  - | 19.6  12.0  4.8 | 571/44 nm  (0.47 0.51) | (*9*) |
| ITO\|HAT-CN (5 nm) \|TAPC (30 nm) \|mCP (10 nm) \|mCBP:BN1 (20 nm) \|POT2T (10 nm) \|TmPYPB (40 nm) \|Liq (1.5 nm) \|Al (100 nm) |  | 3.0  -  - | 6636 | 72.8  -  - | 65.3  -  - | 24.3  18.4  12.9 | 506/36 nm  (0.15, 0.63) | (*10*) |
| ITO\|HAT-CN (5 nm) \|TAPC (30 nm) \|mCP (10 nm) \|mCBP:BN2 (20 nm) \|POT2T (10 nm) \|TmPYPB (40 nm) \|Liq (1.5 nm) \|Al (100 nm) |  | 3.0  -  - | 6286 | 101.6  -  - | 83.1  -  - | 24.5  15.8  7.6 | 545/46 nm  (0.38, 0.61) | (*10*) |
| ITO\|TAPC (30 nm) \|TCTA (10 nm) \|DACT-II (10 nm) :CNCz-BNCz (20 nm) \| TmPYPB (50 nm) \|LiF\|Al |  | 2.7  -  - | - | -  -  - | 117.8  69.1  29.6 | 33.7  27.7  16.4 | 583/49 nm  (0.54, 0.46) | (*10*) |
| ITO\|HATCN (10 nm) \|NPB (30 nm) \| BCzPh(10 nm) \|mCBP:2F-BN (20 nm) \| 9Cz46Pm(10 nm) \|DppyA:Liq(30 nm) \|Liq\|Al |  | 2.6  -  - | - | -  -  - | 69.8  60.1  38.1 | 22.0  20.1  15.0 | 501/40 nm  (0.16, 0.60) | (*11*) |
| ITO\|HATCN (10 nm) \|NPB (30 nm) \| BCzPh(10 nm) \|mCBP:3F-BN (20 nm) \| 9Cz46Pm(10 nm) \|DppyA:Liq(30 nm) \|Liq\|Al |  | 2.6  -  - | - | -  -  - | 72.3  63.1  45.9 | 22.7  22.3  21.1 | 499/38.5 nm  (0.20, 0.58) | (*12*) |
| ITO\|HATCN (10 nm) \|NPB (30 nm) \| BCzPh(10 nm) \|mCBP:4F-BN (20 nm) \| 9Cz46Pm(10 nm) \|DppyA:Liq(30 nm) \|Liq\|Al |  | 2.6  -  - | - | -  -  - | 51.3  42.4  29.4 | 20.9  19.2  16.4 | 493/31.6 nm  (0.12, 0.48) | (*12*) |
| ITO\|TAPC (50 nm) \|TCTA (5 nm) \|PhCzBCz: 15 wt % 5tBuCzBN: 5wt % DtCzB-TPTRZ (30 nm) \|TmPyPB (30 nm) \|LiF (1 nm) \|Al (100 nm) |  | 2.8  -  - | 25500 | 105.8  -  - | 116.2  -  - | 30.6  28.6  16.4 | 520/41 nm  (0.23, 0.68) | (*12*) |
| ITO \|TAPC (50 nm) \|TCTA (5 nm) \|PhCzBCz: 15 wt% sensitizer:3 wt%(R)-OBN-2CN-BN\| TmPyPB (30 nm) \|LiF (1 nm) \|Al (100 nm) |  | 2.8  -  - | 27200 | 71.9  -,  - | 79.8  -,  - | 29.8  27.2  21.2 | 496/33 nm  (0.13, 0.53) | (*13*) |
| ITO \|TAPC (50 nm) \|TCTA (5 nm) \|PhCzBCz:15 wt% sensitizer 3 wt%: (R)-OBN-4CN-BN \| TmPyPB (30 nm) \|LiF (1 nm) \|Al (100 nm) |  | 2.8  -  - | 9052 | 76.1  -,  - | 81.1  -,  - | 24.7  23.5  13.1 | 508/34 nm  (0.16, 0.66) | (*14*) |
| ITO\| HAT-CN (5 nm) \| TAPC (30 nm) \| TCTA (15 nm) \|mCBP(10 nm) \|DMIC-TRZ: 5%BN-CP1(20 nm) PO-T2T(20 nm) \| ANT-BIZ (30 nm) \|LiF (1 nm) \|Al (100 nm) |  | 2.4  -  - | 54753 | 83.8  -  - | 109.7  -  - | 40.0  34.0  18.5 | 496/25 nm  (0.09, 0.50) | (*14*) |
| ITO\| HAT-CN (5 nm) \| TAPC (30 nm) TCTA (15 nm) \| \|mCBP(10 nm)\|DMIC-TRZ: 5%BN-CP2(20 nm) PO-T2T(20 nm) \| ANT-BIZ (30 nm) \|LiF (1 nm) \|Al (100 nm) |  | 2.4  -  - | 57582 | 82.6  -  - | 108.1  -  - | 36.4  32.6  19.2 | 497/26 nm  (0.10, 0.53) | (*15*) |
| ITO\|MoO3\|mcp(50nm)\|DBFDPO:tCBNDA:7 wt % (25nm)\|DBFDPO.40nm\|LiF\|Al |  | 3.5  5.7  8.7 | 4370 | 29.2  18.0  10.2 | 26.2  10.7,  9.4 | 20.2  12.4  7.13 | 472/36 nm  (0.12, 0.17) | This work |
| ITO\|MoO3\|mcp(50nm)\|DBFDPO: tCBNDASPO:20 wt % (25nm)\|DBFDPO.40nm\|LiF\|Al |  | 3.1  5.3  8.1 | 5997 | 40.4  29.8  21.8 | 40.9  17.3  8.6 | 27.9  20.6  15.2 | 472/32 nm  (0.11, 0.17) | This work |

^[a]^Turn-on voltage at 1 cd m^-2^; ^[b]^The maximum luminance; ^[c]^The maximum EL efficiencies.


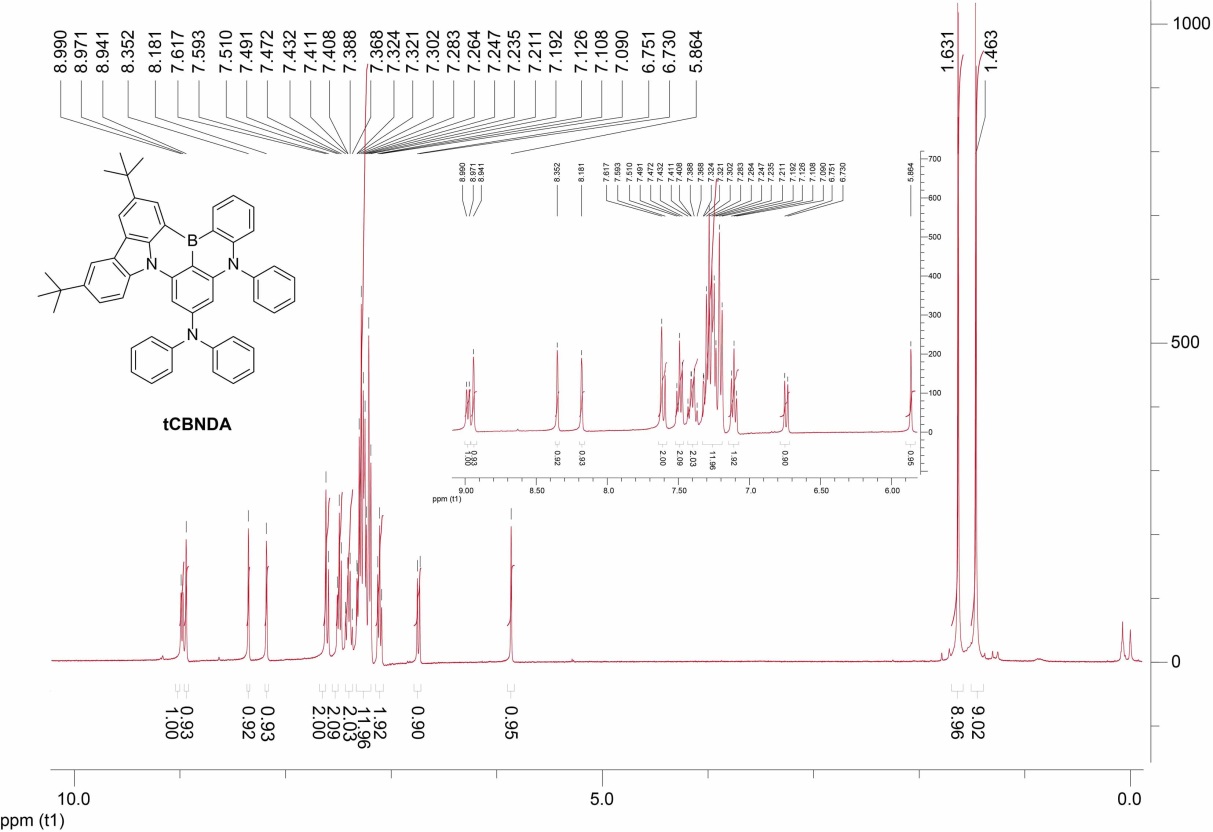


**Figure S22.** ^1^H NMR spectrum of **tCBNDA** in CDCl_3_.


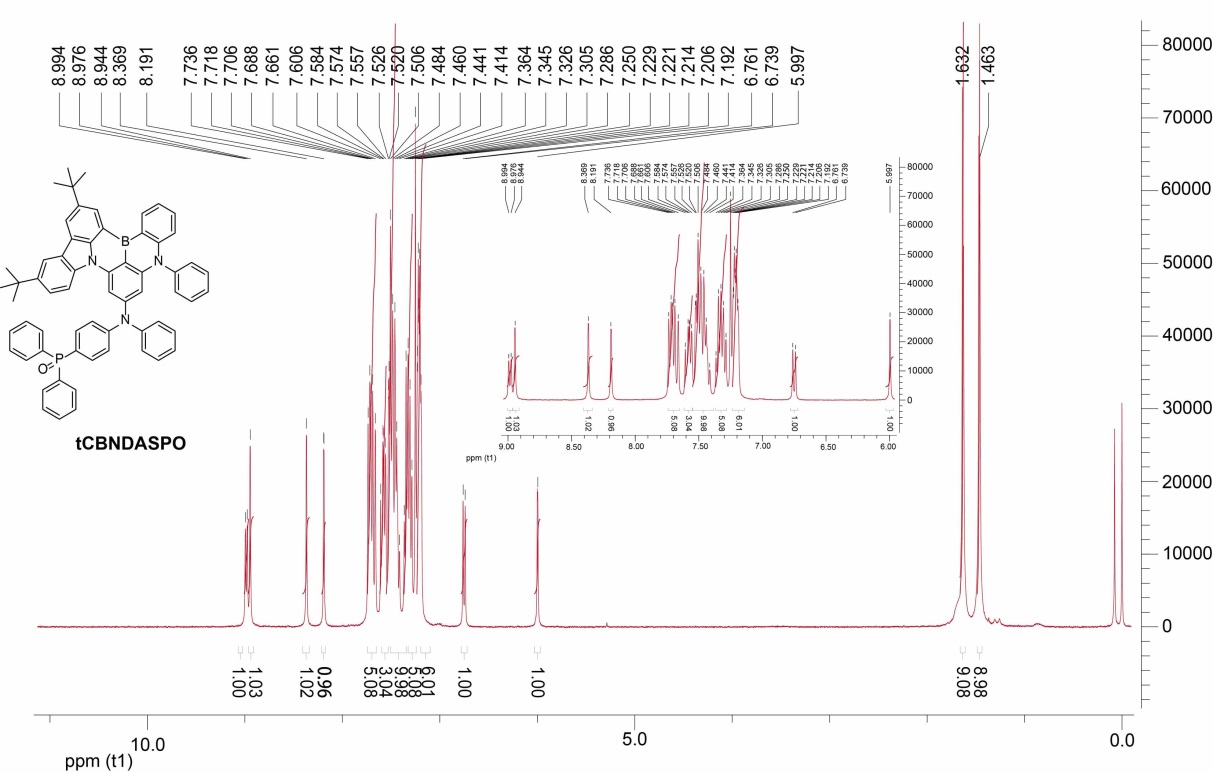


**Figure S23.** ^1^H NMR spectrum of **tCBNDASPO** in CDCl_3_.

^
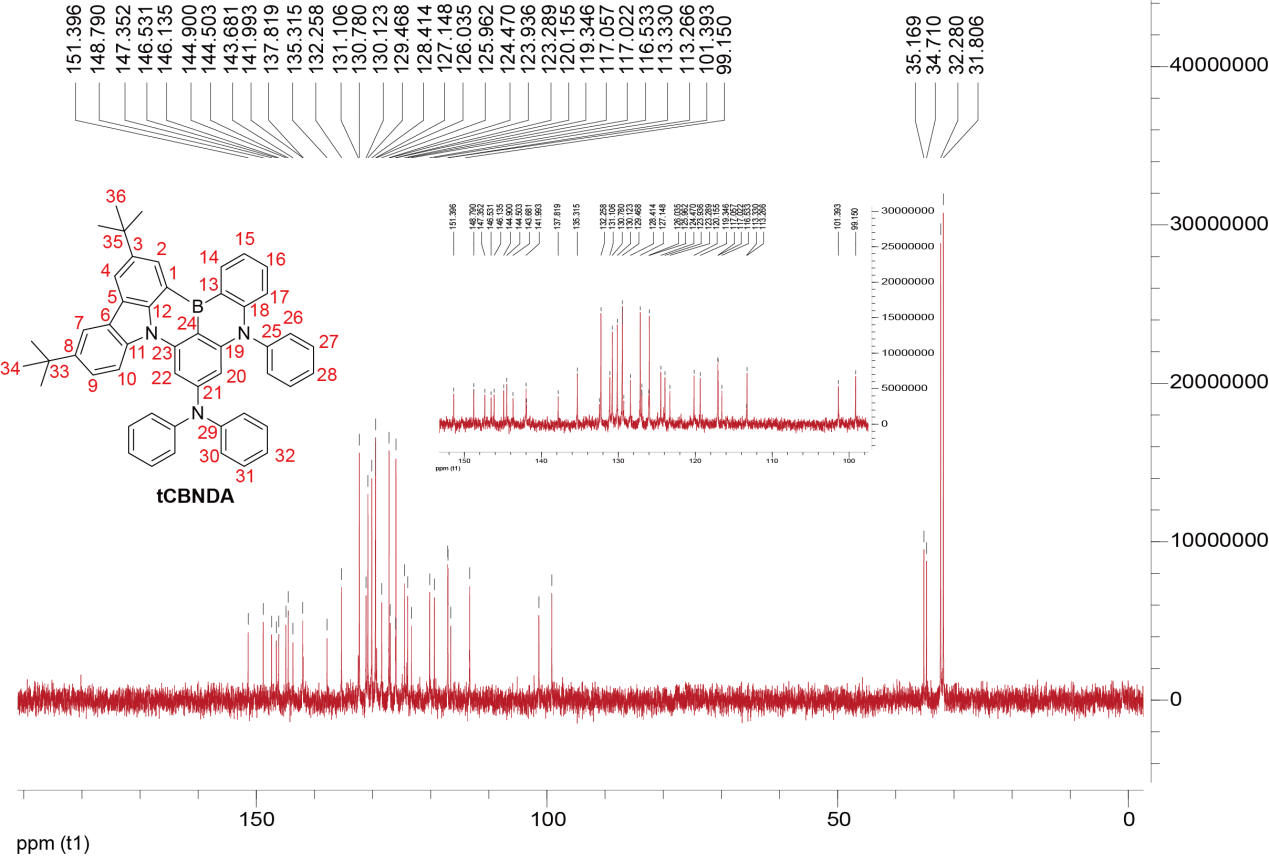
^

**Figure S24.** ^13^C NMR spectrum of **tCBNDA** in CDCl_3_.

**
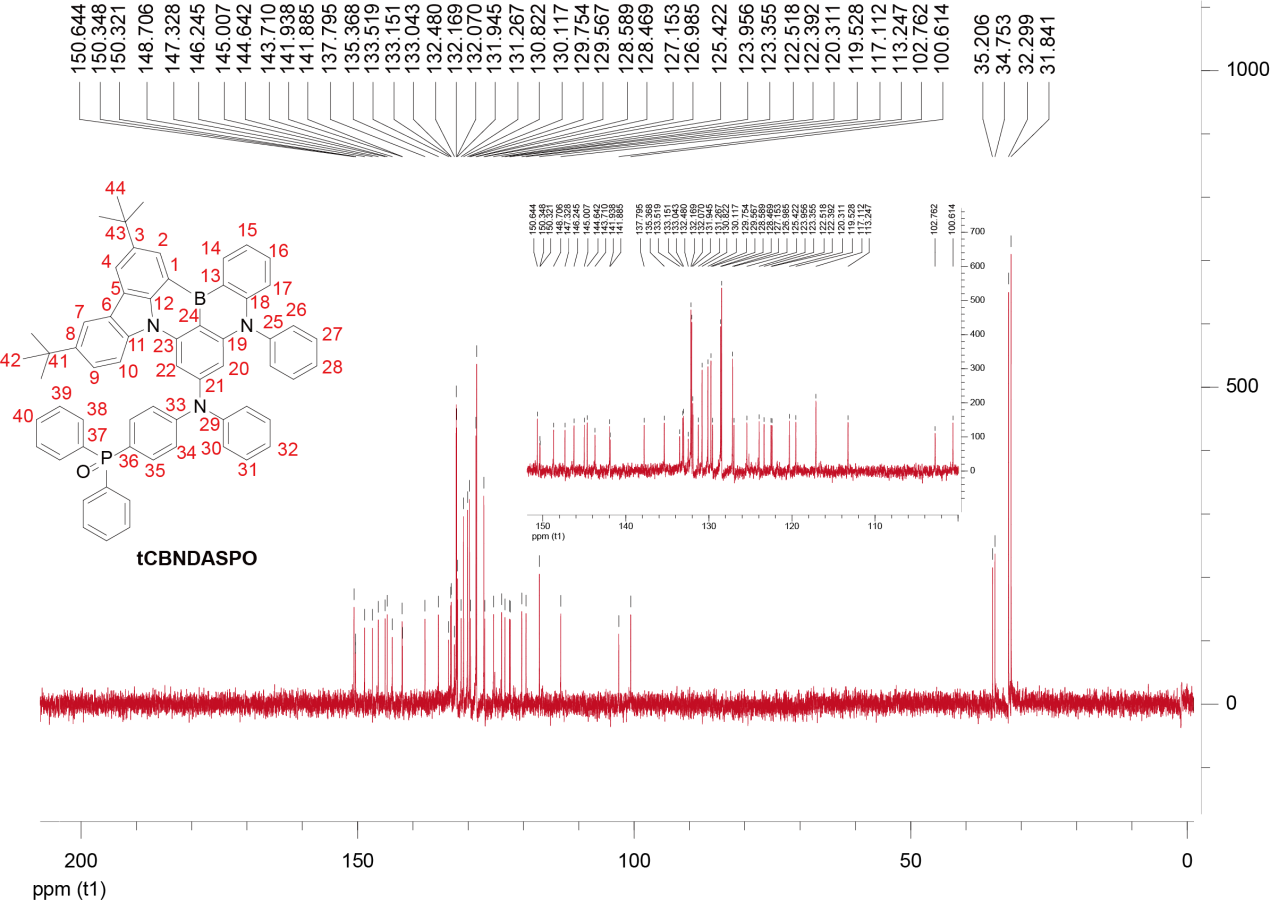
**

**Figure S25.** ^13^C NMR spectrum of **tCBNDASPO** in CDCl_3_.

**
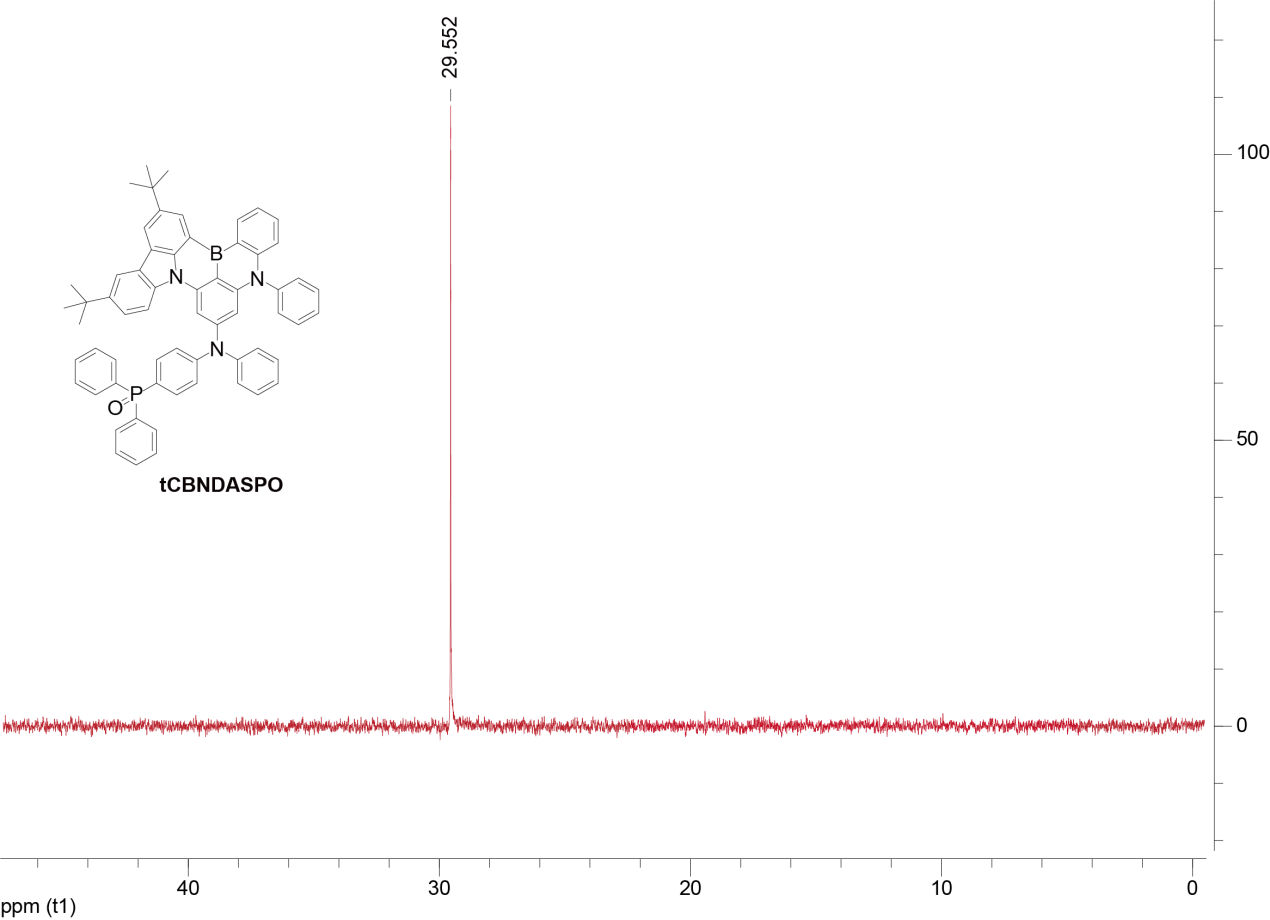
**

**Figure S26.** ^31^P NMR spectrum of **tCBNDASPO** in CDCl_3_.

### VII. References

1. A. D. Becke, Density-functional thermochemistry. III. The role of exact exchange. *J. Chem. Phys.* **98**, 5648-5652 (1993).

2. C. Lee, W. Yang, R. G. Parr, Development of the Colle-Salvetti correlation-energy formula into a functional of the electron density. *Phys. Rev. B* **37**, 785-789 (1988).

3. M. J. Frisch, G. W. Trucks, H. B. Schlegel, G. E. Scuseria, M. A. Robb, J. R. Cheeseman, J. A. Montgomery, J. T. Vreyen, K. N. Kudin, J. C. Burant, J. M. Millam, S. S. Iyengar, J. Tomasi, V. Barone, B. Mennucci, M. Cossi, G. Scalmani, N. Rega, G. A. Petersson, H. Nakatsuji, M. Hada, M. Ehara, K. Toyota, R. Fukuda, J. Hasegawa, M. Ishida, T. Nakajima, Y. Honda, O. Kitao, H. Nakai, M. Klene, X. Li, J. E. Knox, H. P. Hratchian, J. B. Cross, C. Adamo, J. Jaramillo, R. Gomperts, R. E. Stratmann, O. Yazyev, A. J. Austin, R. Cammi, C. Pomelli, J. W. Octhterski, P. Y. Ayala, K. Morokuma, G. A. Voth, P. Salvador, J. J. Dannenberg, V. G. Zakrzewski, S. Dapprich, A. D. Daniels, M. C. Strain, O. Farkas, D. K. Malick, A. D. Rabuck, K. Raghavachari, J. B. Foresman, J. V. Ortiz, Q. Cui, A. G. Baboul, S. Clifford, J. Cioslowski, B. B. Stefanov, A. L. G. Liu, P. Piskorz, I. Komaromi, R. L.Martin, D. J. Fox, T. Keith, M. A. Al-Laham, C. Y. Peng, A. Nanayakkara, M. Challacombe, P. M. W. Gill, B. Johnson, W. Chen, M. W. Wong, C. Gonzalez, J. A. Pople, *Gaussian 03, Revision D.02, Gaussian Inc., Pittsburgh, PA*, ( 2004).

4. Y. Tao, K. Yuan, T. Chen, P. Xu, H. Li, R. Chen, C. Zheng, L. Zhang, W. Huang, Thermally Activated Delayed Fluorescence Materials Towards the Breakthrough of Organoelectronics. *Adv. Mater.* **26**, 7931-7958 (2014)10.1002/adma.201402532).

5. K. Masui, H. Nakanotani, C. Adachi, Analysis of exciton annihilation in high-efficiency sky-blue organic light-emitting diodes with thermally activated delayed fluorescence. *Org. Electron.* **14**, 2721-2726 (2013)10.1016/j.orgel.2013.07.010).

6. X. Liang, Z.-P. Yan, H.-B. Han, Z.-G. Wu, Y.-X. Zheng, H. Meng, J.-L. Zuo, W. Huang, Peripheral Amplification of Multi-Resonance Induced Thermally Activated Delayed Fluorescence for Highly Efficient OLEDs. *Angew.Chem. Int.Ed.* **57**, 11316-11320 (2018)10.1002/anie.201806323).

7. Y. Wang, Y. Duan, R. Guo, S. Ye, K. Di, W. Zhang, S. Zhuang, L. Wang, A periphery cladding strategy to improve the performance of narrowband emitters, achieving deep-blue OLEDs with CIEy < 0.08 and external quantum efficiency approaching 20%. *Org. Electron.* **97**, 106275 (2021) 10.1016/j.orgel.2021.106275).

8. M. Yang, I. S. Park, T. Yasuda, Full-Color, Narrowband, and High-Efficiency Electroluminescence from Boron and Carbazole Embedded Polycyclic Heteroaromatics. *J. Am. Chem. Soc.* **142**, 19468-19472 (2020) 10.1021/jacs.0c10081).

9. M. Yang, S. Shikita, H. Min, I. S. Park, H. Shibata, N. Amanokura, T. Yasuda, Wide-Range Color Tuning of Narrowband Emission in Multi-resonance Organoboron Delayed Fluorescence Materials through Rational Imine/Amine Functionalization. *Angew. Chem. Int. Ed.* **60**, 23142-23147 (2021) 10.1002/anie.202109335).

10. Y. Qi, W. Ning, Y. Zou, X. Cao, S. Gong, C. Yang, Peripheral Decoration of Multi-Resonance Molecules as a Versatile Approach for Simultaneous Long-Wavelength and Narrowband Emission. *Adv. Funct. Mater.* **31**, 2102017 (2021) 10.1002/adfm.202102017).

11. Y. Liu, X. Xiao, Y. Ran, Z. Bin, J. You, Molecular design of thermally activated delayed fluorescent emitters for narrowband orange–red OLEDs boosted by a cyano-functionalization strategy. *Chem. Sci.* **12**, 9408-9412 (2021)10.1039/D1SC02042K).

12. Y. Zhang, D. Zhang, J. Wei, Z. Liu, Y. Lu, L. Duan, Multi-Resonance Induced Thermally Activated Delayed Fluorophores for Narrowband Green OLEDs. *Angew. Chem. Int. Ed.* **58**, 16912-16917 (2019)10.1002/anie.201911266).

13. Y. Xu, C. Li, Z. Li, J. Wang, J. Xue, Q. Wang, X. Cai, Y. Wang, Highly Efficient Electroluminescent Materials with High Color Purity Based on Strong Acceptor Attachment onto B–N-Containing Multiple Resonance Frameworks. *CCS Chem.*, 2077-2091 (2021)10.31635/ccschem.021.202101033).

14. Y. Xu, Q. Wang, X. Cai, C. Li, Y. Wang, Highly Efficient Electroluminescence from Narrowband Green Circularly Polarized Multiple Resonance Thermally Activated Delayed Fluorescence Enantiomers. *Adv. Mater.* **33**, 2100652 (2021)10.1002/adma.202100652).

15. P. Jiang, J. Miao, X. Cao, H. Xia, K. Pan, T. Hua, X. Lv, Z. Huang, Y. Zou, C. Yang, Quenching‐Resistant Multi‐Resonance TADF Emitter Realizes 40% External Quantum Efficiency in Narrowband Electroluminescence at High Doping Level. *Adv. Mater.* **33**, 2106954 (2021)10.1002/adma.202106954).
